# CEDR: robust consensus cancer subtyping with multi-omics data via ensemble dimensionality reduction

**DOI:** 10.1093/bib/bbag232

**Published:** 2026-05-14

**Authors:** Hongyan Cao, Zhaoyang Xu, Shilong Lin, Gang Du, Tong Wang, Juping Wang, Xiaoling Yang, Ruiling Fang, Yanhong Luo, Ping Zeng, Hongmei Yu, Yanbo Zhang, Yuehua Cui

**Affiliations:** Department of Health Statistics, Shanxi Provincial Key Laboratory of Major Diseases Risk Assessment, School of Public Health, Shanxi Medical University, No. 56 South Xinjian Road, Yingze District, Taiyuan, Shanxi 030001, PR China; MOE Key Laboratory of Coal Environmental Pathogenicity and Prevention, Shanxi Medical University, No. 56 South Xinjian Road, Yingze District, Taiyuan, Shanxi 030001, PR China; Department of Health Statistics, Shanxi Provincial Key Laboratory of Major Diseases Risk Assessment, School of Public Health, Shanxi Medical University, No. 56 South Xinjian Road, Yingze District, Taiyuan, Shanxi 030001, PR China; Academy of Medical Sciences, Shanxi Medical University, No. 56 South Xinjian Road, Yingze District, Taiyuan, Shanxi 030001, PR China; Department of Health Statistics, Shanxi Provincial Key Laboratory of Major Diseases Risk Assessment, School of Public Health, Shanxi Medical University, No. 56 South Xinjian Road, Yingze District, Taiyuan, Shanxi 030001, PR China; Department of Health Statistics, Shanxi Provincial Key Laboratory of Major Diseases Risk Assessment, School of Public Health, Shanxi Medical University, No. 56 South Xinjian Road, Yingze District, Taiyuan, Shanxi 030001, PR China; Department of Health Statistics, Shanxi Provincial Key Laboratory of Major Diseases Risk Assessment, School of Public Health, Shanxi Medical University, No. 56 South Xinjian Road, Yingze District, Taiyuan, Shanxi 030001, PR China; Department of Health Statistics, Shanxi Provincial Key Laboratory of Major Diseases Risk Assessment, School of Public Health, Shanxi Medical University, No. 56 South Xinjian Road, Yingze District, Taiyuan, Shanxi 030001, PR China; MOE Key Laboratory of Coal Environmental Pathogenicity and Prevention, Shanxi Medical University, No. 56 South Xinjian Road, Yingze District, Taiyuan, Shanxi 030001, PR China; Department of Thoracic Oncology, Shanxi Bethune Hospital, Shanxi Academy of Medical Sciences, Tongji Shanxi Hospital, Third Hospital of Shanxi Medical University, No. 99 Longcheng Street, Xiaodian District, Taiyuan, Shanxi 030032, PR China; Department of Health Statistics, Shanxi Provincial Key Laboratory of Major Diseases Risk Assessment, School of Public Health, Shanxi Medical University, No. 56 South Xinjian Road, Yingze District, Taiyuan, Shanxi 030001, PR China; MOE Key Laboratory of Coal Environmental Pathogenicity and Prevention, Shanxi Medical University, No. 56 South Xinjian Road, Yingze District, Taiyuan, Shanxi 030001, PR China; Department of Health Statistics, Shanxi Provincial Key Laboratory of Major Diseases Risk Assessment, School of Public Health, Shanxi Medical University, No. 56 South Xinjian Road, Yingze District, Taiyuan, Shanxi 030001, PR China; MOE Key Laboratory of Coal Environmental Pathogenicity and Prevention, Shanxi Medical University, No. 56 South Xinjian Road, Yingze District, Taiyuan, Shanxi 030001, PR China; Department of Biostatistics, School of Public Health, Xuzhou Medical University, No. 209 Tongshan Road, Yunlong District, Xuzhou, Jiangsu 221004, PR China; Department of Health Statistics, Shanxi Provincial Key Laboratory of Major Diseases Risk Assessment, School of Public Health, Shanxi Medical University, No. 56 South Xinjian Road, Yingze District, Taiyuan, Shanxi 030001, PR China; MOE Key Laboratory of Coal Environmental Pathogenicity and Prevention, Shanxi Medical University, No. 56 South Xinjian Road, Yingze District, Taiyuan, Shanxi 030001, PR China; Department of Health Statistics, Shanxi Provincial Key Laboratory of Major Diseases Risk Assessment, School of Public Health, Shanxi Medical University, No. 56 South Xinjian Road, Yingze District, Taiyuan, Shanxi 030001, PR China; MOE Key Laboratory of Coal Environmental Pathogenicity and Prevention, Shanxi Medical University, No. 56 South Xinjian Road, Yingze District, Taiyuan, Shanxi 030001, PR China; Department of Statistics and Probability, Michigan State University, 619 Red Cedar Road, East Lansing, MI 48824, United States

**Keywords:** multi-omics integration, dimension reduction, clustering ensemble, cancer subtyping, robust clustering

## Abstract

Cancer is a highly heterogeneous disease underpinned by complex molecular alterations. Accurate subtyping is critical for guiding personalized treatment and improving clinical outcomes. However, multi-omics data are high-dimensional, noisy, and heterogeneous across platforms, posing major challenges for reliable subtyping. To address this, dimensionality reduction is necessary to capture underlying molecular patterns in a low-dimensional space, facilitating both computational efficiency and biological interpretation. We present Consensus subtyping method with Ensemble Dimensionality Reduction for multi-omics data integration (CEDR), a consensus subtyping framework that integrates complementary linear and nonlinear dimensionality reduction methods with robust clustering and probabilistic ensemble modeling. Different from existing dimensionality reduction techniques, our framework adopts an ensemble learning framework that integrates multiple dimensionality reduction techniques with robust clustering to achieve reliable consensus cancer subtyping. We apply Optimally Tuned Robust Improper Maximum Likelihood Estimator to the concatenated low-dimensional matrix for robust subtyping, and ensemble the result with the Mixture Model for Clustering Ensembles to identify stable subtypes. Across extensive simulations, CEDR consistently outperformed conventional dimensionality reduction-based clustering, the Cluster Of Clusters Analysis (COCA) ensemble strategy, and state-of-the-art multi-omics integration algorithms (SNF and CIMLR) in both accuracy and robustness. Application to clear cell renal cell carcinoma and lower-grade glioma revealed biologically interpretable subtypes characterized by distinctive survival outcomes, pathway activities, and immune infiltration patterns. These findings demonstrate that CEDR provides a powerful and reliable strategy for multi-omics data integration and cancer subtyping, with strong potential for broader applications in high-dimensional multimodal data analysis.

## Graphical Abstract

**Graphical Abstract:**
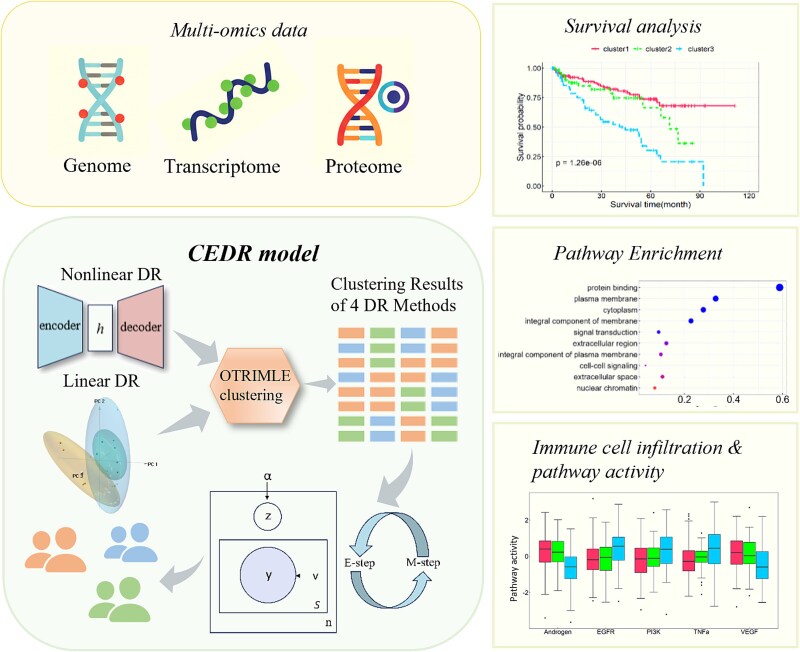

## Introduction

Cancer is a highly heterogeneous disease, as tumors originating from the same tissue and sharing identical histological grades and pathological stages may display distinct molecular mechanisms across patients [1, 2]. Defining molecular subtypes through shared features and uncovering subtype-specific alterations linked to clinical outcomes are crucial for targeted therapies, improved prognosis, and personalized treatment strategies.

With the development of high-throughput technology, a large amount of omics data has been accumulated, and it is crucial to use the integration of multi-omics data to achieve accurate subtyping of cancer patients [3]. Nevertheless, multi-omics integration faces key challenges: (i) the small number of samples relative to the high dimensionality of each omics layer; (ii) differences in scale, noise, and batch effects across datasets; and (iii) the complementary yet heterogeneous nature of information across omics data types. Therefore, numerous methods for integrating multi-omics data have been proposed [4, 5]. Early integration represents a straightforward approach by concatenating omic matrices into a single matrix and applying single-omic clustering. However, it has drawbacks, including potential bias toward omics with more features, neglect of different data distributions, and increased dimensionality [4]. Late integration involves clustering each omic independently, with the resulting clustering solutions combined to produce a unified clustering outcome. However, relying solely on clustering solutions during integration may overlook weak signals present in individual omics. Statistical modeling integrates all omics data by modeling the probabilistic distribution, often incorporating biological knowledge through Bayesian priors or probabilistic functions, though parameter estimation can be computationally challenging, often requiring heuristics. Sample similarity integration relies on similarities or distances between samples to perform clustering, but interpreting results in terms of original features can be difficult. Dimension reduction-based method assumes an inherent low-dimensional structure, often consistent with cluster numbers, enabling precise cancer patient subtyping by mapping data into a unified low-dimensional space, interpreting dominant cluster features, and mitigating the “curse of dimensionality” to enhance pattern extraction while reducing noise and redundancy [6–9].

Dimensionality reduction methods can be broadly categorized into linear and nonlinear approaches [10]. Linear methods, such as Principal Component Analysis (PCA) [11], Independent Component Analysis (ICA) [12], and Non-negative Matrix Factorization (NMF) [13], are widely used for feature extraction in high-dimensional biological data. However, linear methods rely on linear assumptions, limiting their ability to capture the complex, nonlinear interactions between genes and their self-regulation [14]. Consequently, nonlinear techniques including t-Distributed Stochastic Neighbor Embedding (t-SNE) [15], Uniform manifold approximation and projection (UMAP) [16], Isomap [17], and kernel PCA [18] have been developed to better preserve nonlinear structures. With the development of deep learning technology, Autoencoder (AE) compresses high-dimensional data into a lower-dimensional subspace [19], enabling the extraction of nonlinear patterns from high-dimensional data. However, AE is highly sensitive to noise in the input data, and due to its lack of robustness, it becomes challenging to extract the most informative features from high-dimensional multi-omics data in practical applications. In contrast, advanced AE models, such as Denoising Autoencoder (DAE) [20] and Sparse Autoencoder (SAE) [21], offer improved robustness and feature extraction capabilities, effectively addressing these limitations. Dimensionality reduction methods are often applied to each omics dataset separately, and the resulting features are then concatenated into a single matrix for clustering. To further address the challenges of outlier handling and clustering robustness, the Optimally Tuned Robust Improper Maximum Likelihood Estimator (OTRIMLE) clustering approach [22] can be adopted. This method introduces a noise component to capture outliers and noise, optimizing its value to ensure that the non-noise portion of the model closely approximates a Gaussian mixture distribution, thereby achieving robust clustering for high-noise and anomalous data [23].

However, different dimensionality reduction methods-based clustering results rely on distinct assumptions and data characteristics, leading to inconsistent identification of cancer subtypes across methods. One intuitive strategy to overcome this issue is the development of ensemble methods that integrate results from multiple base methods [24].

In this paper, we employ a statistically grounded consensus clustering model, the Mixture Model for Clustering Ensembles [25]. This multinomial mixture framework effectively manages inconsistent cluster numbers, mismatched cluster labels across methods, and missing labels in some solutions. By combining the strengths of dimensionality reduction, OTRIMLE, and Mixture Model for Clustering Ensembles, we propose a robust **C**onsensus subtyping method with **E**nsemble **D**imensionality **R**eduction for multi-omics data integration (CEDR). CEDR employs four dimensionality reduction techniques for multi-omics data, including DAE, SAE, PCA, and ICA, to generate diverse and informative feature representations, respectively. OTRIMLE is applied to each representation to obtain robust subtyping results. These results are then integrated using the Mixture Model for Clustering Ensembles, producing a consensus subtyping solution with improved robustness and consistency.

We conducted simulation studies to evaluate the subtyping performance of the proposed CEDR method by comparing it with clustering approaches based on DAE, SAE, PCA, and ICA, as well as the consensus clustering ensemble method Cluster Of Clusters Analysis (COCA) [26, 27] based on the same base clustering methods, and state-of-the-art integration methods such as SNF [28], CIMLR [29], LSGMC [30], MOSD [31], and PartLES [32]. We further applied the CEDR method to two real-world datasets, Clear Cell Renal Cell Carcinoma (ccRCC) and Lower-Grade Glioma (LGG), obtained from The Cancer Genome Atlas (TCGA) and the Chinese Glioma Genome Atlas (CGGA), respectively. The results indicate that CEDR outperforms other methods, consistent with the simulation results, in identifying molecular subtypes of ccRCC and LGG patients. The subsequent biological analyses of key molecular features and pathways provide valuable mechanistic insights into the two cancers.

## Methods

### The overview of CEDR

**Figure 1:**
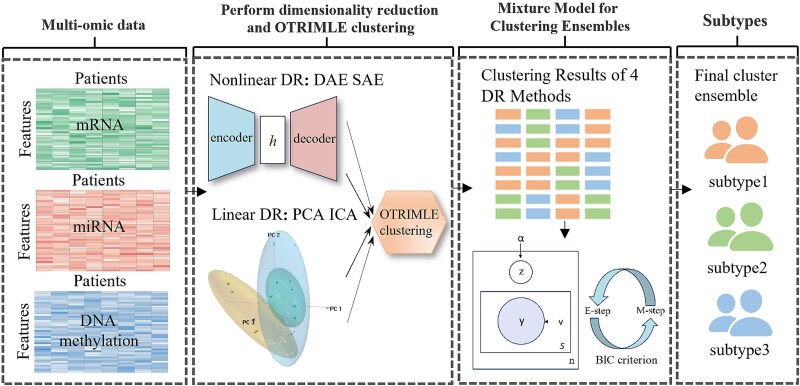
Overview of the CEDR model. The CEDR workflow integrates three omics data types, including miRNA, mRNA, and DNA methylation, and applies four dimensionality reduction techniques (DAE, SAE, PCA, and ICA) to extract informative features. OTRIMLE is then used to perform robust clustering on the dimensionality-reduced data, generating multiple base clusterings that serve as inputs to a mixture model–based ensemble framework. Within this ensemble, the EM algorithm is employed to perform maximum likelihood estimation with the BIC guiding the selection of the optimal number of consensus clusters, ultimately yielding a consensus clustering that stratifies patients into distinct subtypes.

An overview of the CEDR method is depicted in Fig. 1. CEDR takes multi-omics data matrices as input and employs a comprehensive strategy to improve cancer subtyping by integrating multiple dimensionality reduction–based clustering results within a mixture model framework, combined with robust clustering techniques. Specifically, CEDR performs subtyping through a three-stage process:

(1) *Dimensionality reduction:* Four dimensionality reduction methods (DAE, SAE, PCA, and ICA) are used to reduce data dimensionality and mitigate noise, each capturing unique structural features of the data.
(2) *OTRIMLE clustering:* Based on the concatenated feature matrices obtained from each dimensionality reduction method, OTRIMLE is applied to generate robust clustering results that capture complementary subtyping structures across multi-omics data.
(3) *Mixture Model for Clustering Ensembles:* The multiple subtyping results obtained from Step 2 are further integrated using the Mixture Model for Clustering Ensembles, yielding a consensus subtyping solution with enhanced robustness and consistency. The Mixture Model for Clustering Ensembles is a probabilistic ensemble framework based on a mixture of multivariate multinomial distributions [19], from which the final consensus subtyping solution is derived by solving a maximum likelihood problem using the expectation–maximization (EM) algorithm. In the following sections, we provide a detailed description of the implementation.

### Three-stage implementation framework

CEDR is implemented through a three-stage framework, as detailed below.

#### Stage 1: Dimensionality reduction

CEDR begins by applying four complementary dimensionality reduction techniques to multi-omics data, namely two nonlinear deep learning–based methods, denoising autoencoder (DAE) and sparse autoencoder (SAE), and two linear techniques, principal component analysis (PCA) and independent component analysis (ICA). These methods aim to mitigate noise, reduce dimensionality, and capture diverse structural patterns from different omics modalities. For illustration, we describe the DAE-based dimensionality reduction process below, while implementation details of the remaining methods are provided in the Supplementary Note 1.

Let ${A}_k\in{R}^{n\times{p}_k}$ denote the $k$th ($k=1,\cdots, K$) omics data matrix with $n$ samples and ${p}_k$ features, each ${A}_k$ serving as the input to an autoencoder. The hidden layer is configured with ${m}_k$ nodes, where ${m}_k<{p}_k$, to perform dimensionality reduction. Compressed features are then extracted from the bottleneck layer and represented as matrices ${H}_k$ of dimensions $n\times{m}_k$ for each omics type.

Unlike the traditional AE, DAE generates partially corrupted data by introducing noise into the input, then reconstructs the original input through an encoding-decoding process [6]. This enhances the deep neural network’s capacity to discern salient features. The corrupted input, denoted as $\overset{\sim }{x}$, is expressed as:


(1) \begin{equation*} \overset{\sim }{x}\sim{q}_D\left(\overset{\sim }{x}\mid x\right), \end{equation*}

where ${q}_D\left(\overset{\sim }{x}\mid x\right)$ denotes a stochastic corruption process that injects noise into the original input $x$. The corrupted input $\overset{\sim }{x}$ is then fed into a deep neural network for encoding and decoding, following the same process as a standard autoencoder. The loss function of DAE is expressed as:


(2) \begin{equation*} {J}_{DAE}\left(W,b\right)=\sum{E}_{\overset{\sim }{x}\sim{q}_D\left(\overset{\sim }{x}|x\right)}\left[L\left(x,y\right)\right], \end{equation*}

where $x$ and $y$ are the original and reconstructed inputs, respectively, and $L\left(x,y\right)$ denotes the squared error loss.

We employ the ReLU, $f(x)=\mathit{\max}\left(0,x\right)$, as the nonlinear activation function in both DAE and SAE. Finally, the ${L}_1$ and ${L}_2$ regularizations were added to the model to improve the generalization ability and robustness of the autoencoder. The autoencoder was implemented through the R software h2o package. Further details on SAE, PCA, and ICA, including parameter configurations, are provided in the Supplementary Note 1.

#### Stage 2: OTRIMLE clustering

Based on the low-dimensional features ${H}_k$ obtained in Stage 1, we integrated the reduced features from the three omics datasets $\left(\mathrm{with}\ K=3,\mathrm{for}\ \mathrm{example}\right)$. For each dimensionality reduction method (DAE, SAE, PCA, and ICA), the corresponding components were concatenated to yield an integrated feature matrix *H* of dimension $n\times \left({m}_1+{m}_2+{m}_3\right)$, where ${m}_k$ denotes the reduced dimensionality of the $k$-$th$ omics dataset$.$The OTRIMLE method was then applied to the integrated matrix *H* to perform clustering and achieve robust subtyping.

Let the number of target clusters be $G$, then OTRIMLE will look for $G$ classes that satisfy the elliptically symmetric shape in these $n$ samples. The main idea of the robust improper maximum likelihood estimator (RIMLE) is to employ a pseudo-model in which noise is represented by an improper constant density defined over the entire Euclidean space. Let $n$ samples be *H*$=\{{h}_1,{h}_2,\dots, {h}_n\}$, and let $\phi \left(h;{\mu}_j,{\Sigma}_j\right)$ be the Gaussian density at the point $h$ centered on the mean vector ${\mu}_j$ with a covariance matrix ${\Sigma}_j$. The shape of the $j$th group is characterized by the level sets of let $\phi \left(\cdot; {\mu}_j,{\Sigma}_j\right)$, $j=1,2,\dots, G$. Assume that the expected proportion of each group is set as ${\pi}_j\in \left[0,1\right]$, $\sum_{j=1}^G{\pi}_j=1-{\pi}_0$, where ${\pi}_0$ denotes the proportion of all points that are independent of the symmetric shapes of the $G$ ellipses, which are noises and outliers, and $0\le{\pi}_0<1$. The sample distribution is modeled by the following pseudo-mixture density:


(3) \begin{equation*} {\psi}_{\delta}\left(h;\theta \right)={\pi}_0\delta +\sum_{j=1}^G{\pi}_j\phi \left(h;{\mu}_j,{\Sigma}_j\right) \end{equation*}

where $\delta$ is the noise component, which can accommodate even arbitrarily extreme outliers. $\theta$ is the parameter vector containing all ${\pi}_j,{\mu}_j,{\Sigma}_j$, and ${\pi}_0$. Equation (3) is designed for multivariate continuous data, where meaningful clusters can be reasonably approximated by elliptical shapes. However, the model does not require exact Gaussianity. The improper constant density component (${\pi}_0\delta$) provides built-in robustness by capturing observations that deviate from the Gaussian prototype, including heavy-tailed distributions, arbitrary noise, and isolated outliers, which makes the procedure particularly well suited for noisy multi-omics datasets. The RIMLE is given by the following constrained optimization problem:


(4) \begin{equation*} {\displaystyle \begin{array}{c}\kern3.25em \mathit{\max}\sum_{i=1}^n\log{\psi}_{\delta}\left({h}_i;\theta \right)\kern8em \\{}\mathrm{subject}\ \mathrm{to}\ 0\le{\pi}_j\le 1,\mathrm{for}\ \mathrm{all}\ j=0,1,\cdots, G\end{array}} \end{equation*}

$$ {\displaystyle \begin{array}{c}\frac{\lambda_{max}\left(\theta \right)}{\lambda_{min}\left(\theta \right)}\le \gamma \kern4em \\{}\sum_{i=1}^n\frac{\pi_0\delta }{\psi_{\delta}\left({h}_i;\theta \right)}\le n{\pi}_{max}\end{array}} $$

where ${\lambda}_{max}\left(\theta \right)$ and ${\lambda}_{min}\left(\theta \right)$denote the maximum and minimum eigenvalues, respectively, calculated over all fractional covariance matrices in $\theta$. $\gamma \ge 1$, and ${\pi}_{max}\in \left(0,1\right)$ denotes the maximum proportion of noise and outliers.

The optimization of the above problem depends on the $\delta$ value, and RIMLE can be computed on a grid of $\delta$ values ranging from zero to a sufficiently large $\delta$ value to find the best $\delta$ value, which ultimately yields the optimally robust pseudo-maximum likelihood estimator OTRIMLE. Let ${\theta}^{\ast }$ be the solution of OTRIMLE, the samples are assigned based on the following rule:


(5) \begin{equation*} J\left({h}_i,{\theta}^{\ast}\right)= argma{x}_{j\epsilon \left\{0,1,2,\dots, G\right\}}{\tau}_j\left({h}_i,{\theta}^{\ast}\right) \end{equation*}

where ${\tau}_j\left({h}_i,{\theta}^{\ast}\right)=\frac{\pi_j^{\ast}\phi \left({h}_i,{\mu}_j^{\ast },{\Sigma}_j^{\ast}\right)}{\psi_{\delta^{\ast }}\left({h}_i,{\theta}^{\ast}\right)}$,$\mathrm{if}\ j=1,\dots, G$, and ${\tau}_0\left({h}_i,{\theta}^{\ast}\right)=\frac{\pi_0{\delta}^{\ast }}{\psi_{\delta^{\ast }}\left({h}_i,{\theta}^{\ast}\right)}$, $\mathrm{if}\ j=0$ corresponds to noise.

Let ${\pi}_l\left({h}_i\right)$ denote the clustering assignment obtained by OTRIMLE under the $l$-$th$ dimensionality reduction method, where each sample ${h}_i$ is assigned to one of the $G$ identified subtypes or classified as noise/outliers $\left(j=0,1,\dots, G\right)$. Accordingly, each sample ${h}_i$ is subjected to OTRIMLE-based subtyping across $S=4$ distinct feature spaces derived from four dimensionality reduction techniques, resulting in a set of subtype assignments: $\left\{{\pi}_1\left({h}_i\right),{\pi}_2\left({h}_i\right),\dots, {\pi}_S\left({h}_i\right)\right\}$.

#### Stage 3: Mixture model for clustering ensembles

For the $n$ samples, given these multiple base clustering results $\left\{{\pi}_1\left({h}_i\right),{\pi}_2\left({h}_i\right),\dots, {\pi}_S\left({h}_i\right)\right\}$ obtained in Stage 2 ($i=1,2,\dots, n$), we further ensemble the results using a mixture model-based ensemble approach to obtain a more robust consensus partition. Let ${y}_{il}={\pi}_l\left({h}_i\right)$, and ${y}_i=\left\{{y}_{i1},\cdots, {y}_{iS}\right\}=\pi \left({h}_i\right)$ represent the cluster assignment vector for sample ${h}_i$. The set $Y=\left\{{y}_1,\cdots, {y}_n\right\}$ is used to construct the consensus partition ${\pi}_C$. We model ${y}_i$ as random variables drawn from a mixture of $Q$ multivariate densities, where $Q$ is the number of consensus clusters, each parameterized by (${\theta}_q$, $q=1,2,\dots, Q$), with prior probabilities ${\alpha}_q$. The probability is defined as $P\left({y}_i|\varTheta \right)=\sum_{q=1}^Q{\alpha}_q{P}_q\left({y}_i|{\theta}_q\right)$. All the data $Y={\left\{{y}_i\right\}}_i^n$ are assumed to be independent and identical distribution, the log-likelihood is:


(6) \begin{equation*} \log L\left(\varTheta |Y\right)=\log \prod_{i=1}^nP\left({y}_i|\varTheta \right)=\sum_{i=1}^n\mathit{\log}\sum_{q=1}^Q{\alpha}_q{P}_q\left({y}_i|{\theta}_q\right) \end{equation*}

The optimal parameters are obtained by maximizing (6), that is:


(7) \begin{equation*} {\varTheta}^{\ast }=\mathit{\arg}{\mathit{\max}}_{\varTheta}\log L\left(\varTheta |Y\right) \end{equation*}

To simplify the modeling, the components of vector ${y}_i$ are assumed to be conditionally independent, namely that the conditional probability of ${y}_i$ can be represented as the following product [25]:


(8) \begin{equation*} {P}_q\left({y}_i|{\theta}_q\right)=\prod_{l=1}^S\kern0.1em \prod_{j=1}^{G(l)}\kern0.1em {\nu}_{lq}{(j)}^{\delta \left({y}_{il},j\right)} \end{equation*}

where $G(l)$ denotes the number of clusters in the $l$-$th$ base partition ${\pi}_l$, ${\nu}_{lq}(j)$ represents the probability that a sample in the $q$-$th$ consensus cluster is assigned to cluster $j$ in the $l$-$th$ base partition, satisfying: $\sum_{j=1}^{G(l)}{v}_{lq}(j)=1$. To provide further justification, it is worth noting that even if the different clustering algorithms (indexed by $l$, $l=1,2,\dots, S$) are not strictly independent, the product approximation in Eq. (8) is still supported by the strong empirical performance of naive Bayes classifiers in discrete domains [25].

The optimization of the likelihood in Eq. (7) can use the EM algorithm. Assuming the existence of latent data $Z$, the likelihood of the complete data $\left(Y,Z\right)$ can be written as $\log L\left(\varTheta |Y,Z\right)=\mathit{\log}\prod_{i=1}^nP\left({y}_i,{z}_i|\varTheta \right)\kern0.1em$. In the *E*-Step, it computes the expected values of the latent variables $E\left[{z}_{iq}\right]$, and in the *M*-Step, it updates the parameters by maximizing the expected log-likelihood. Parameters are initialized using a Dirichlet prior with a uniform parameter vector (all elements set to 1) and iterated until the log-likelihood difference between consecutive iterations is <0.0001. The consensus partition ${\pi}_C$ is then derived by assigning each sample ${z}_i$ to the cluster $m$ with the highest posterior probability: ${\pi}_C\left({z}_i\right)=\arg \underset{q\in \left\{1,\dots, Q\right\}}{\mathit{\max}}\kern0.1em E\left[{z}_{iq}\right]$.

The number of consensus clusters $Q$ was then determined by the Bayesian Information Criterion (BIC), where the model with the lowest BIC was selected as the optimal solution, balancing model fit and complexity.

### Determining the optimal cluster number for base clustering

For each base clustering derived from different dimensionality reduction techniques, we determined the optimal number of clusters using the Calinski–Harabasz (CH) index [33], defined as:


(9) \begin{equation*} CH=\frac{\sum_{j=1}^c\kern0.1em {n}_j\parallel{\mu}_j-\mu{\parallel}^2/\left(c-1\right)}{\sum_{j=1}^c\kern0.1em \sum_{i=1}^{n_j}\parallel{h}_i-{\mu}_j{\parallel}^2/\left(n-c\right)} \end{equation*}

where $c$ is the number of clusters, $\mu$ is the overall mean, ${\mu}_j$ is the mean of the $j$-$th$ cluster, ${n}_j$ is the number of samples in the $j$-$th$ cluster, and ${h}_i$ denotes the $i$-$th$ sample. The function calculates the ratio of intercluster distance to intracluster distance, and the larger the ratio, the better the clustering effect.

### Simulation study

To evaluate the robustness and performance of the CEDR method in subtype identification based on multi-omics data, we conducted simulation studies to compare CEDR with dimensionality reduction-based clustering methods (DAE, SAE, PCA, and ICA), the COCA ensemble method [26, 27] applied to the same base clustering, and other multi-omics clustering algorithms SNF [28], CIMLR [29], LSGMC [30], MOSD [31], and PartLES [32].

Following the settings outlined in the literature [34, 35], we constructed three types of omics data, each dataset comprising 200 samples and 1000 features. These 200 samples were evenly distributed across four subtypes, with 50 samples per subtype. To configure a predefined clustering structure for the simulated data matrix. By configuring the three omics datasets: ${X}_i={mean}^s+{\varepsilon}_i$, where ${mean}^s$ represents the average expression level of signal features in each dataset, and $\varepsilon \sim N\ \left(0,{\sigma}^2\right)$ represents Gaussian noise randomly added to the dataset. Specifically, samples 1–50, 51–150, and 151–200 in ${X}_1$ with${mean}^s\in \left\{1,0,3\right\}$; samples 1–50/101–150, 51–100, and 151–200 in ${X}_2$ with ${mean}^s\in \left\{0,2,3\right\}$; samples 1–100, 101–150, and 151–200 in ${X}_3$ with ${mean}^s\in \left\{2,1,3\right\}$. We increase the difficulty of clustering the simulated datasets. Three sets of datasets, named SimData1 (Signal% = 6%), SimData2 (Signal% = 8%), and SimData3 (Signal% = 10%), are established with varying proportions of signal features. Three different noise levels are set: low, moderate, and high (${\sigma}^2=2$, ${\sigma}^2=4$, and ${\sigma}^2=8$). A low proportion of signal features and high variance are expected to reduce clustering accuracy, making it more difficult to distinguish the four clusters. To thoroughly assess the robustness and consistency of CEDR under varying conditions, each scenario was simulated 1000 times.

We use Normalized Mutual Information (NMI) [36] to assess the clustering performance of different methods. NMI measures the similarity and consistency between the true and estimated clusters, with values ranging from 0 to 1. A higher NMI indicates better clustering performance. It is defined as:


$$ NMI=\frac{\mathrm{I}\left(\mathrm{U},\mathrm{V}\right)}{\sqrt{\mathrm{H}\left(\mathrm{U}\right)\mathrm{H}\left(\mathrm{V}\right)}}, $$

where $U$ and $V$ represent the true and estimated clusters, respectively, $\mathrm{I}\left(\mathrm{U},\mathrm{V}\right)$ is their mutual information, and $\mathrm{H}\left(\cdot \right)$ is the entropy function.

### Real data analysis

#### Real datasets and data processing

We focused on two independent cancer data: ccRCC and LGG. The ccRCC data were obtained from the TCGA database using the TCGAbiolinks R package [37], while the LGG data were obtained from the CGGA database [38]. Detailed datasets and data processing for ccRCC and LGG are provided in the supplementary files (Supplementary Note 2). After preprocessing, we retained 388 miRNAs, 16 893 mRNAs, and 10 994 promoter CpG methylation features for 285 ccRCC patients, and 827 miRNAs, 19 416 mRNAs, and 14 470 methylated genes for 86 LGG patients. The baseline characteristics are shown in Supplementary Table S1.

#### Downstream statistical analysis after subtyping

##### Survival analysis

We analyzed survival differences among patients with different subtypes using Kaplan–Meier survival curves and the log-rank test [39]. To assess the prognostic value of the subtyping results while adjusting for tumor grade (pathological stage), age, and sex, we employed the Cox proportional hazards model.

##### Differential and enrichment analysis

To further elucidate molecular heterogeneity and confirm the biological significance of the subtypes, we identified differentially expressed miRNAs (DEmiRNAs), mRNAs (DEmRNAs), and differentially methylated genes (DMGs) using the Kruskal–Wallis *H*-test [40], with a significance threshold of FDR-adjusted *P*-value < .05. Enriched genes in each subtype were further identified with the hypergeometric distribution test [29], using a filtering criterion of $({P}_{adj}<.05 )$. The target genes of DEmiRNAs were predicted via the miRWalk online platform [41] and subsequently integrated with DEmRNAs and DMGs for joint analysis. The overlapping genes across different omics datasets were identified and visualized using a Venn diagram. Kyoto Encyclopedia of Genes and Genomes (KEGG) [42] and Gene Ontology (GO) [43] enrichment analyses of the overlapping genes were performed using the KOBAS 3.0 online tool [44].

##### Immune cell infiltration analysis and pathway activity

Tumor cell composition was estimated using the TIMER2 online tool [45], and immune infiltrating cells with significant differences among subtypes were identified via the Kruskal–Wallis *H*-test. Additionally, pathway activity was computed using the PROGENy R package [46].

## Results

### Simulation results

We summarized the NMI values from different clustering methods in Table 1. The NMI value of CEDR is overall higher than those of the dimensionality reduction-based methods (DAE, SAE, PCA, and ICA), the COCA ensemble strategy, and the state-of-the-art integration methods SNF, CIMLR, LSGMC, MOSD, and PartIES, demonstrating the accuracy and robustness of CEDR in subtyping. As the proportion of signal features increases and the noise variance decreases, the performance of most methods improves. It also indicates that the ensemble strategy achieves higher NMI values than SNF and CIMLR. Under a 6% signal strength and high noise variance (σ^2^ = 8), the NMI value for CEDR is 0.778, compared with 0.648 for COCA, 0.323 for SNF, and 0.346 for CIMLR. Notably, the deep learning–based DAE and SAE methods outperform linear dimensionality reduction–based clustering methods, as well as SNF and CIMLR, demonstrating the particular advantages of deep learning for dimensionality reduction. Moreover, the standard deviation of the NMI values for CEDR is the lowest across all conditions, demonstrating that CEDR is the most robust method for subtyping. In addition to NMI, we further compared clustering performance using Adjusted Rand Index (ARI), Adjusted Mutual Information (AMI), and clustering accuracy (ACC), with the results summarized in (Supplementary Note 3, Supplementary Tables S2–S4, which consistently support the superior robustness and accuracy of CEDR.

**Table 1:** Clustering performance of CEDR and other methods assessed by NMI.

**Method**	**SimData1 (Signal% = 6%)**			**SimData2 (Signal% = 8%)**			**SimData3 (Signal% = 10%)**		
	**Low** **noise**	**Moderate** **noise**	**High** **noise**	**Low** **noise**	**Moderate** **noise**	**High** **noise**	**Low** **noise**	**Moderate** **noise**	**High** **noise**
**CEDR**	**0.991** **(0.021)**	**0.939** **(0.033)**	**0.778** **(0.058)**	**0.997** **(0.016)**	**0.974** **(0.026)**	**0.862** **(0.049)**	**0.999** **(0.011)**	**0.985** **(0.029)**	**0.924** **(0.038)**
DAE-based	0.986 (0.056)	0.939 (0.048)	0.750 (0.086)	0.979 (0.068)	0.973 (0.052)	0.862 (0.066)	0.981 (0.058)	0.970 (0.078)	0.924 (0.056)
SAE-based	0.988 (0.032)	0.919 (0.056)	0.708 (0.099)	0.993 (0.042)	0.969 (0.027)	0.827 (0.083)	0.993 (0.049)	0.978 (0.044)	0.910 (0.053)
PCA-based	0.795 (0.103)	0.670 (0.107)	0.570 (0.047)	0.860 (0.088)	0.733 (0.114)	0.605 (0.064)	0.904 (0.074)	0.796 (0.101)	0.655 (0.101)
ICA-based	0.481 (0.051)	0.492 (0.055)	0.493 (0.060)	0.475 (0.049)	0.485 (0.052)	0.493 (0.056)	0.474 (0.052)	0.482 (0.052)	0.489 (0.055)
COCA	0.887 (0.108)	0.800 (0.094)	0.648 (0.110)	0.908 (0.104)	0.843 (0.108)	0.725 (0.111)	0.912 (0.101)	0.885 (0.111)	0.784 (0.102)
SNF	0.581 (0.050)	0.428 (0.043)	0.323 (0.037)	0.723 (0.061)	0.513 (0.045)	0.374 (0.038)	0.851 (0.049)	0.593 (0.051)	0.417 (0.041)
CIMLR	0.697 (0.054)	0.501 (0.049)	0.346 (0.042)	0.828 (0.049)	0.621 (0.052)	0.421 (0.038)	0.916 (0.034)	0.713 (0.052)	0.488 (0.045)
LSGMC	0.673 (0.048)	0.533 (0.045)	0.391 (0.04)	0.761 (0.06)	0.602 (0.042)	0.462 (0.045)	0.827 (0.054)	0.662 (0.05)	0.517 (0.045)
MOSD	0.541 (0.052)	0.383 (0.043)	0.383 (0.043)	0.671 (0.05)	0.47 (0.051)	0.309 (0.044)	0.762 (0.05)	0.566 (0.053)	0.368 (0.042)
PartIES	0.896 (0.028)	0.777 (0.033)	0.503 (0.164)	0.941 (0.025)	0.842 (0.03)	0.842 (0.03)	0.967 (0.022)	0.884 (0.029)	0.753 (0.038)

Note: The NMI values are presented as the mean (standard deviation) of 1000 simulation runs. The best-performing result is highlighted in bold.

Overall, CEDR exhibits high consensus robustness and outperforms current state-of-the-art methods in clustering accuracy.

### Overall subtyping performance of ccRCC and LGG

**Figure 2:**
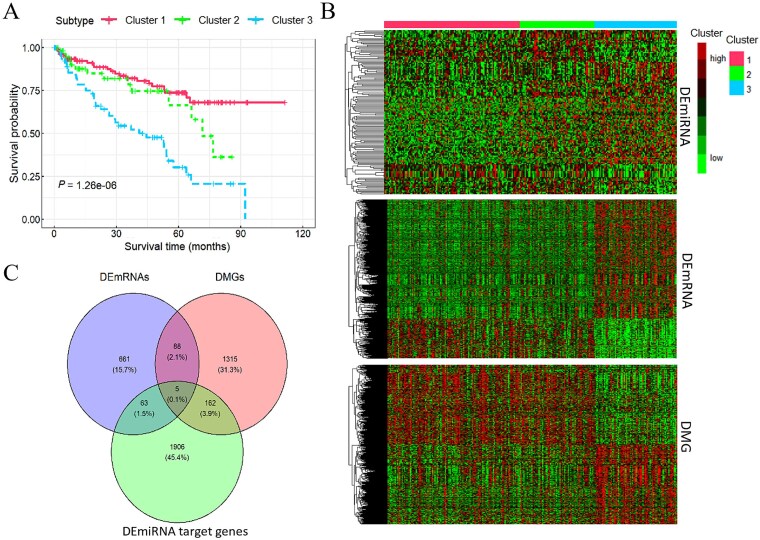
Subtyping results of ccRCC. (A) Kaplan–Meier curves of subtypes identified by CEDR for ccRCC. (B) The heatmaps of DEmRNAs, DEmiRNAs, and DMGs between different clusters. (C) Venn diagram of differential genes in ccRCC patients.

We compared the performance of CEDR with other methods in cancer subtyping using two cancer datasets, ccRCC and LGG. The optimal number of clusters for CEDR was determined using the BIC. To ensure comparability, the optimal number of clusters for the COCA ensemble strategy, SNF, and CIMLR was set to the same as CEDR, whereas for the dimensionality reduction–based clustering methods (DAE, SAE, PCA, and ICA), it was determined using the CH index. CEDR optimally classified ccRCC patients into three subtypes and LGG patients into two subtypes (see Table 2). Log-rank test results for the identified subtypes demonstrated that CEDR outperformed other methods, as indicated by the smallest *P*-values. This suggests that the subtypes identified by CEDR exhibit more significant survival differences than those derived from the other seven methods. Furthermore, in most instances, clustering outcomes derived from deep learning–based autoencoders outperformed those obtained through PCA and ICA dimensionality reduction techniques, emphasizing the superior capabilities of deep learning autoencoders in dimensionality reduction. We also observed that Kaplan–Meier curves constructed based on CEDR-defined subtypes showed clearer separation between subtypes, revealing stronger prognostic differences (Fig. 2A and Supplementary Fig. S3A).

**Table 2:** Comparison of subtyping results of different methods for ccRCC and LGG.

Method	ccRCC		LGG	
	Number of clusters	Log rank *P*-value	Number of clusters	Log rank *P*-value
CEDR	3	**1.26E-06**	2	**2.94E-11**
DAE	3	3.17E-05	3	1.51E-08
SAE	2	4.75E-05	2	3.17E-09
PCA	2	1.70E-04	3	3.56E-07
ICA	4	3.28E-05	4	1.38E-07
COCA	3	6.36E-06	2	5.56E-06
SNF	3	1.62E-04	2	1.29E-07
CIMLR	3	4.21E-02	2	0.793

Note: The “log rank *P*-value” refers to the *P*-value obtained from the log-rank test. Among the results, CEDR achieves the smallest prognostic P-value, which is highlighted in bold font.

### Analysis of ccRCC subtypes identified by CEDR

Using CEDR, we classified 248 ccRCC patients into three subtypes with significant prognostic differences (115 patients in Cluster 1, 63 in Cluster 2, and 70 in Cluster 3) (see Fig. 2A, ${\chi}^2=27.2$, log-rank *P*-value = 1.26 × 10^−6^). Thirty-seven ccRCC patients were trimmed as outliers during OTRIMLE clustering to ensure the robustness of subtype identification. Detailed clinical and pathological characteristics and survival outcomes for these outlier patients are provided in Supplementary Note 4 (Figure S1–S2) and Table S5. The relevant clinical characteristics of each cluster are summarized in Supplementary Table S5, where Cluster 3 exhibited poorer prognosis and a higher proportion of patients with advanced pathological stages.

After adjusting for covariates including pathological stage, age, and gender, we fitted a multivariable Cox proportional hazards model using the three subtypes as independent variables and survival status (survival or death) and survival time as dependent variables, with results summarized in Supplementary Table S6. Patients in Cluster 3 exhibited a 2.48-fold higher risk of death compared to those in Cluster 1. Similarly, patients with pathological Stage III and Stage IV had a 3.558-fold and 7.482-fold higher risk of death, respectively, compared to those with Stage I.

### Significant feature identification in ccRCC

Based on the clustering results of CEDR for ccRCC patients, we conducted differential expression (DE) analysis to identify significant features across the identified subtypes. This analysis identified 817 DEmRNAs, all of which were upregulated, along with 1570 DMGs (including 978 hypomethylated and 592 hypermethylated genes) and 135 DEmiRNAs (69 downregulated and 66 upregulated). Heatmaps illustrating DE across different omics data are presented in Fig. 2B, where each row represents a distinct feature and each column corresponds to a patient. In these heatmaps, red and green colors denote relatively high and low expression levels, respectively, highlighting substantial heterogeneity among the three subtypes. A total of 318 overlapping genes were identified for ccRCC (Fig. 2C).

### GO and KEGG pathway enrichment analysis in ccRCC

**Figure 3:**
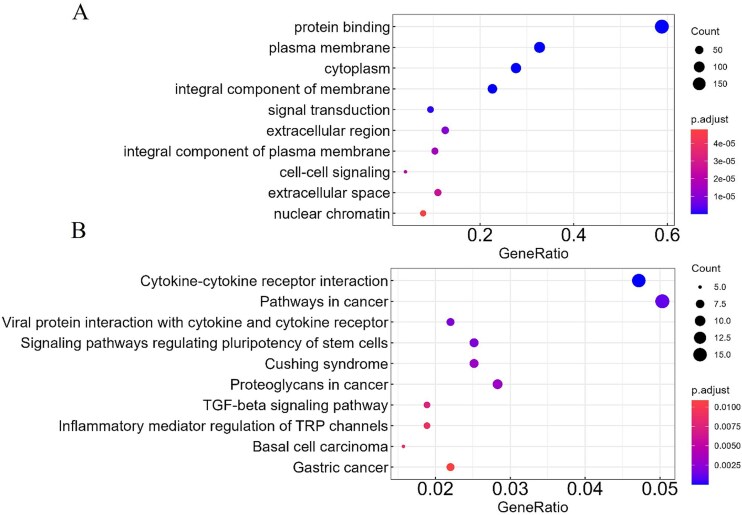
Enrichment analysis of overlapping genes in ccRCC. (A) GO biological process enrichment and (B) KEGG pathway enrichment for 318 genes. The size of the circle represents the number of genes (Count) enriched in each biological function.

We conducted GO and KEGG pathway enrichment analyses on the resulting overlapping genes to gain insight into their functional characteristics. For ccRCC, the top 10 enriched GO biological process terms and KEGG pathways are shown in Fig. 3. The GO terms, mainly belonging to the cellular component and molecular function categories, were enriched in plasma membrane, integral component of plasma membrane, extracellular region, extracellular space, cytoplasm, nuclear chromatin, protein binding, signal transduction, and cell–cell signaling. Some studies have shown that some SARS-CoV-2 protein-binding human mRNAs (SPBRs) are related to the occurrence and development of ccRCC, confirming that it is an important biomarker [47]. The KEGG pathways were significantly enriched in cytokine–cytokine receptor interaction, pathways in cancer, viral protein interaction with cytokine and cytokine receptor, signaling pathways regulating pluripotency of stem cells, Cushing syndrome, proteoglycans in cancer, Transforming growth factor beta (TGF-beta) signaling pathway, inflammatory mediator regulation of TRP channels, basal cell carcinoma, and gastric cancer. Among these, the cytokine–cytokine receptor interaction pathway has been identified as a key prognostic pathway in ccRCC [48, 49].

### Immune cell infiltration analysis and pathway activity in ccRCC

**Figure 4:**
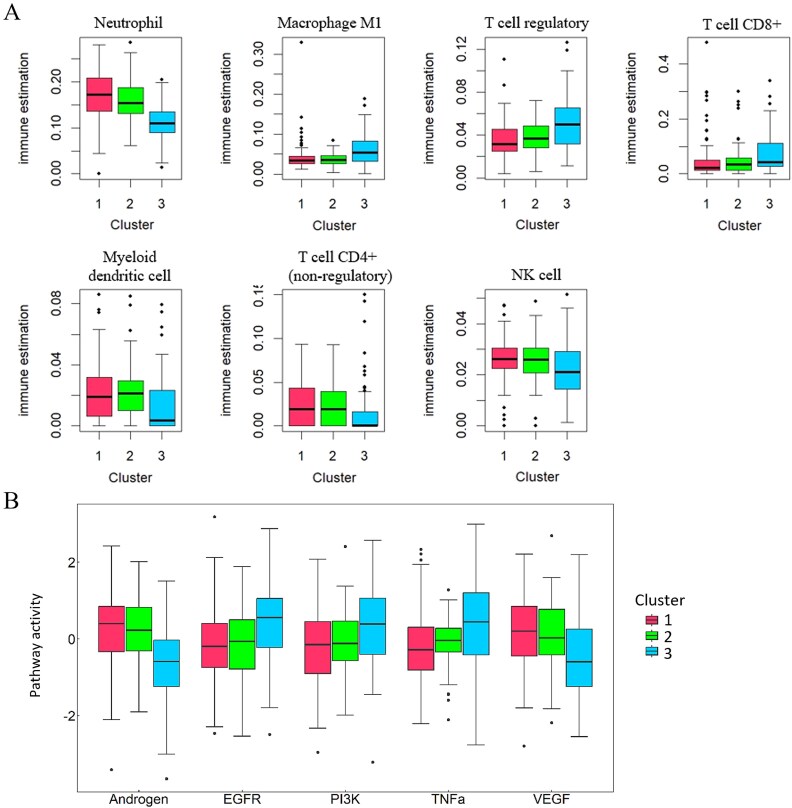
Differences in pathway activity and immune cell infiltration across clusters in ccRCC. (A) Abundance of Neutrophil, Macrophage M1, T cell regulatory, T cell CD8^+^, Myeloid dendritic cell, T cell CD4^+^ (non-regulatory), and NK cell. (B) Pathway activity of Androgen, EGFR, PI3K, TNFα, and VEGF across three clusters in ccRCC.

To further elucidate the biological and clinical significance of the identified subtypes in ccRCC, we conducted analyses of immune cell infiltration and pathway activity. The immune cell infiltration diagram for three subtypes of ccRCC patients is illustrated in Fig. 4A. We identified seven immune infiltrating cell types exhibiting significant differences across the three groups, including seven immune cells [Neutrophil, Macrophage M1, T cell regulatory, T cell CD8+, Myeloid dendritic cell, T cell CD4+ (nonregulatory), and natural killer (NK) cell]. Clearly, the three subtypes display significantly different abundances of Neutrophil, Macrophage M1, T cell regulatory, T cell CD8+, Myeloid dendritic cell, T cell CD4+ (non-regulatory), and NK cell. Cluster 3 represents the subtype with the poorest prognosis, and the tumor microenvironment of Cluster 3 is characterized by high infiltration of Macrophage M1, T cell regulatory, and T cell CD8+, while the abundance of Neutrophil, Myeloid dendritic cell, T cell CD4+ (nonregulatory), and NK cell is relatively low. Previous studies have shown that neutrophil extracellular traps (NETs) serve as a robust biomarker for patient stratification and treatment decisions in ccRCC and may potentially influence the tumor microenvironment dynamics of its subtypes [50]. CTLA-4 and PD-1 are key regulators of T-cell function and play a crucial role in tumor immune tolerance. Targeting these molecules has been shown to enhance antibody efficacy in antitumor immunity, offering new strategies for clinical therapy [51].

We identified the five most significant pathways, as shown in Fig. 4B. We observed that in Cluster 3, which exhibits the worst overall survival rate, the activity of the EGFR, PI3K, and TNFα pathways is significantly higher compared to the other two clusters, whereas the androgen and vascular endothelial growth factor (VEGF) pathways show the highest activity in Cluster 1. It has been shown that androgen receptors (ARs) bind to androgen response elements (AREs) in the promoter, upregulating TANAR and promoting vasculogenic mimicry (VM) formation, thereby driving ccRCC progression and metastasis [52]. VEGF enhances vascular permeability and fibrin accumulation, sustaining tumor growth and invasion, while its expression serves as a marker of malignancy and metastatic potential [53]. Additionally, CAV2 silencing suppresses EGFR/PI3K expression, suggesting that targeting this pathway holds therapeutic promise for ccRCC [54]. The difference in pathway activity between the three subtypes further reflects the heterogeneity among different subtypes of ccRCC.

### Robustness and adaptability of CEDR to other cancer subtyping

We implemented similar analysis strategies for the LGG data and identified two subtypes by CEDR. These subtypes were significantly associated with overall survival (Supplementary Fig. S3A, log-rank *P*-value = 2.94 × 10^−11^). After adjusting for age, gender, and tumor grade, Cluster 2 patients had a 7.724-fold higher risk of death than Cluster 1 patients (*P-*value = .008) in the Cox regression analysis (Supplementary Table S8). Using the same analytical framework, CEDR stratified 262 patients from the TCGA Liver Hepatocellular Carcinoma (LIHC) cohort into two molecular subtypes with significantly different overall survival outcomes (Supplementary Fig. S6, log-rank *P*-value = 3.47 × 10^−2^). Twenty-five LIHC patients were identified as outliers and excluded to enhance the robustness of subtype identification during OTRIMLE clustering. Due to space limitations, the details of the LGG and LIHC subtype analysis results are provided in the supplementary file (Supplementary Note 5, Figure S3–S5, Table S7–S8 for LGG and Supplementary Note 6, Figure S6 for LIHC).

### Sensitivity analysis and ablation study

Sensitivity analyses were conducted for the DAE and SAE components to evaluate the robustness of the framework with respect to variations in the corruption rate and sparsity regularization strength. In addition, we performed an ablation study to assess the contribution of OTRIMLE to overall robustness. OTRIMLE consistently outperformed *k*-means and spectral clustering, demonstrating superior stability and effective modeling of DR-derived feature spaces. Detailed results are provided in the online Supplemental Material (Supplementary Note 7, Figure S7–S9).

## Discussion

Cancer is a complex and highly heterogeneous disease. Accurately identifying cancer subtypes is essential for uncovering potential therapeutic targets and achieving precision medicine. We proposed a consensus ensemble dimension reduction-based robust clustering method for cancer subtyping using multi-omics data, CEDR, which addresses the challenges of high dimensionality noise and outliers that often compromise the robustness of cancer subtyping. By ensembling multiple dimensionality reduction techniques, it effectively mitigates these issues, enhancing the accuracy and consistency of subtype identification. Extensive simulations and applications to LGG and ccRCC datasets demonstrate that CEDR outperforms existing integrative subtyping methods, providing a powerful and reliable tool for robust cancer subtyping.

Our approach is novel in several aspects. First, it integrates multiple dimensionality reduction techniques, including nonlinear deep learning-based autoencoders (DAE and SAE) and linear methods (PCA and ICA), to extract informative features from multi-omics data, effectively denoising the data and enhancing the consistency of downstream clustering results. Second, the highly adaptive OTRIMLE effectively handles noise and outliers, ensuring reliable cluster identification even in the presence of data contamination. By employing the highly adaptive OTRIMLE for base clustering on the dimensionality-reduced features, followed by ensembling via a Mixture Model for Clustering Ensembles, CEDR achieves robust subtype identification. Third, the nonlinear feature extraction capability of DAE and SAE enables CEDR to capture complex patterns in multi-omics data, yielding superior subtyping performance compared to using linear methods alone. Together, these steps create an ensemble-based framework that enhances the consistency and robustness of clustering results across omics layers by systematically mitigating noise and outliers, further supported by our comprehensive simulation studies.

In applications to two distinct cancer types, ccRCC and LGG, CEDR identified biologically meaningful subtypes after extensive downstream statistical and bioinformatics analyses. Compared to subtypes obtained using base clustering and other integrative methods, the subtypes discovered by CEDR exhibited significant differences in gene enrichment, cancer-related biological pathways, and immune cell infiltration. For instance, ccRCC patients were stratified into three subtypes. Patients in Cluster 3 exhibited a significantly elevated mortality risk relative to the other two subtypes, indicating that this subtype may correspond to a clinically aggressive disease phenotype, in concordance with results reported in previous studies [55]. Furthermore, this association remained statistically significant after adjusting for age, gender, and pathological stage in multivariable Cox regression analysis. In our analysis of the LGG cohort, Cluster 2 showed a relatively higher proportion of Tumor grade III cases and correspondingly worse survival outcomes. This concordance between tumor grade and the prognosis of CEDR-defined subtypes provides biological support for the validity of the consensus subtyping results. These findings further demonstrate the advantages of CEDR in integrative subtyping and provide valuable biological insights for clinical decision-making.

Similar to other studies, this work has several limitations that should be acknowledged. First, CEDR is an unsupervised subtyping framework. As an exploratory approach, the subtypes identified by unsupervised methods are not derived from clinical outcomes but are instead examined for their clinical relevance. Moreover, we demonstrated the application of CEDR on two cancer datasets, ccRCC and LGG. Benchmarking its performance on additional datasets is desirable and will be pursued in future work. In addition, while representative dimensionality reduction methods were selected in this study, systematically exploring other combinations of methods may further improve the robustness of the framework and will be considered in future work. Beyond these aspects, emerging computational technologies and biological resources also offer opportunities for further improvement. For example, recent advances in large language models (LLMs) for biomedical data analysis have shown promising capabilities in multi-omics integration, feature interpretation, and cross-modal reasoning, which may enhance the explainability and knowledge integration of subtype discovery frameworks [56]. Moreover, curated biological resources such as CircR2Cancer [57] provide experimentally supported associations between circular RNAs and cancers, and integrating such prior biological knowledge could facilitate the interpretation and validation of subtype-specific molecular signatures. Finally, our method focuses on integrating bulk multi-omics datasets. Future work will explore integrative approaches for single-cell multi-omics data, such as scMNMF [58] and GSTRPCA [59].

In summary, we propose CEDR as an innovative strategy for integrating multi-omics data, specifically designed to address the high dimensionality, noise, and presence of outliers inherent in such data. CEDR enables robust subtyping of LGG and ccRCC patients, achieving higher subtype identification accuracy compared to base clustering methods and other state-of-the-art approaches. By facilitating patient stratification, CEDR supports precise treatment and early intervention, which are critical for preventing cancer metastasis and progression. Beyond its application to multi-omics bulk data, CEDR is also applicable to unsupervised subtyping in single-cell multi-omics data and other multimodal datasets.

**Key Points**

- CEDR introduces a novel consensus subtyping framework that integrates multiple dimensionality reduction techniques (DAE, SAE, PCA, ICA) with robust clustering (OTRIMLE) and a mixture-model ensemble to achieve stable and accurate multi-omics cancer subtyping.
- Through extensive simulations, CEDR consistently outperforms state-of-the-art methods, including SNF, CIMLR, COCA, and single-method dimensionality reduction approaches, in both clustering accuracy and robustness under varying signal and noise conditions.
- Applied to real ccRCC and LGG datasets, CEDR identifies biologically meaningful subtypes with strong survival differences, uncovers key subtype-specific biomarkers, and reveals distinct pathway and immune infiltration patterns.
- CEDR provides a general and powerful strategy for multi-omics data integration, offering broad applicability to cancer subtyping and other unsupervised tasks in high-dimensional multi-modal data analysis.

## Supplementary Material

CEDR_supplementary_materials_BIB_final_bbag232(1)

## Data Availability

The ccRCC and LIHC data analyzed in this study can be accessed through the Genomic Data Commons Data Portal (https://www.cancer.gov/ccg/research/genome-sequencing/tcga). The LGG data analyzed are available in the CGGA (http://www.cgga.org.cn/).

## Software availability

The source code for CEDR is available at https://github.com/biostatXu/CEDR.
