# Supplementary material for: CEDR: robust consensus cancer subtyping with multi-omics data via ensemble dimensionality reduction
Source: Brief Bioinform. 2026 May 14;27(3):bbag232. doi: 10.1093/bib/bbag232 (PMC13174402; doi:10.1093/bib/bbag232)
Supplement: CEDR_supplementary_materials_BIB_final_bbag232(1) [file cedr_supplementary_materials_bib_final_bbag232(1).docx]

^[[1]](#footnote-1)^ Supplementary Materials for **“****CEDR: Consensus robust cancer subtyping with multi-omics data via** **ensemble dimension reduction”**

Hongyan Cao^a,b*^, Zhaoyang Xu^a,c^, Shilong Lin^a,b^, Gang Du^a^, Tong Wang^a,c^, Juping Wang^a,b^, Xiaoling Yang^d^, Ruiling Fang^a,b^, Yanhong Luo^a,b^, Ping Zeng^e^, Hongmei Yu^a,b^, Yanbo Zhang^a,b^, Yuehua Cui^f*^

^a^*Department of Health Statistics*, *Shanxi Provincial Key Laboratory of Major Diseases Risk Assessment*, *School of Public Health*, *Shanxi Medical University*, *Taiyuan*, *Shanxi 030001*, *PR China*

^b^*MOE Key Laboratory of Coal Environmental Pathogenicity and Prevention*, *Shanxi Medical University*, *Taiyuan*, *Shanxi 030001*, *PR China.*

^c^*Academy of Medical Sciences*, *Shanxi Medical University*, *Taiyuan, Shanxi 030001*, *PR China*

^d^*Department of Thoracic Oncology*, *Shanxi Bethune Hospital, Shanxi Academy of Medical Sciences*, *Tongji Shanxi Hospital*, *Third Hospital of Shanxi Medical University*, *Taiyuan*, *Shanxi 030032*, *PR China*

^e^*Department of Biostatistics*, *School of Public Health, Xuzhou Medical University*, *Xuzhou*, *Jiangsu 221004*, *PR China*

^f^*Department of Statistics and Probability, Michigan State University, East Lansing, MI 48824, USA*

**1. Supplementary** **Note 1: Dimensionality reduction**

**1.1 Dimensionality reduction methods**

We employed four complementary dimensionality reduction techniques on multi-omics data, including two advanced autoencoder (AE) models, denoising autoencoder (DAE) and sparse autoencoder (SAE), and two linear techniques, principal component analysis (PCA) and independent component analysis (ICA).

The Autoencoder (AE) is a neural network designed for dimensionality reduction and noise reduction through a lossy compression process [[1](#_ENREF_20)]. It trains an encoder-decoder structure, consisting of an input layer, hidden layer, and output layer, to reconstruct the input $x\in R^{n}$ as output $y$, minimizing the reconstruction error$\left\| y-x \right\|_{2}^{2}$ using a loss function $J_{AE}\left( W,b \right)=\sum L\left( x,y \right)$. The encoder $h=T\left( W_{1}x+b_{1} \right)$ maps input to a lower-dimensional hidden representation, while the decoder $y=T\left( W_{2}h+b_{2} \right)$ reconstructs it, with $W_{1}$, $b_{1}$ and $W_{2}$, $b_{2}$ as weights and biases, and $T$ as a nonlinear activation function (e.g., Sigmoid, ReLU). and when the number of nodes in the hidden layer is smaller than the number of nodes in the input layer, the dimensionality reduction and noise reduction of the data are realized.

The sparse autoencoder (SAE) is an advanced extension of the traditional autoencoder. It addresses the issue of learning ineffective features, which can occur when the hidden layer contains too many nodes, by introducing sparsity penalties to suppress the activation of certain neurons. This not only prevents overfitting but also enhances the efficiency of the autoencoder.

The loss function of the SAE is defined as:

$J_{SAE}\left( W,b \right)=\sum\left( L\left( x,y \right) \right)+\beta\sum_{j=1}^{h} KL(\rho||\hat{\rho}_{j})$ (1)

where $\beta$ is a coefficient, typically in the range $\left[ 0,1 \right]$, that adjusts the weight of the sparsity penalty term; $KL(\rho||\hat{\rho}_{j})$ represents the Kullback-Leibler (KL) divergence, which enforces sparsity by penalizing deviations between the target sparsity $\rho$ and the average activation $\hat{\rho}_{j}$; $\hat{\rho}_{j}=\frac{1}{u}\sum_{i=1}^{u} \left( a_{j}\left( x_{i} \right) \right)$ denotes the average activation of the $j$-th neuron in the hidden layer over $u$ input samples; $a_{j}\left( x_{i} \right)$ is the activation of the $j$-th hidden neuron for the $i$-th input sample.

For the classical feature extraction technique principal component analysis (PCA), an orthogonal transformation is used to covert a set of possibly correlated variables into a set of linearly uncorrelated components, reducing high-dimensional data to a lower-dimensional space while retaining most of the original information [2], and effectively reducing redundancy and noise. The detailed theory of PCA is omitted here for brevity.

Independent Component Analysis (ICA) is a statistical method used for blind source separation to isolate independent components from a mixed signal. The basic idea is to decompose a mixed signal into uncorrelated source signals such that these components are statistically independent. ICA assumes that the mixed signal matrix$X=(x_{1},x_{2},\cdots,x_{n})^{T}$ is an *n*-dimensional random observation vector, which is obtained by linearly mixing *n* unknown independent components $S=(s_{1},s_{2},\cdots,s_{n})^{T}$, and assumes that the source signals are always independent of each other and non-Gaussian distributed, then the expression of this mixing model is:

$X=AS=\sum_{j=1}^{n} a_{j}s_{j}$ (2)

where $j=1,2,\cdots n,$ $A=\left( a_{1},a_{2},\cdots,a_{n} \right)$ is an $n\times n$ order full rank constant matrix and is the mixing matrix of the source signal.

**1.2 Parameter configurations**

The optimal number of hidden layer nodes in the autoencoder for methylation, mRNA, and miRNA data were determined to be 10, 10, and 90 for LGG, and 10, 10, and 60 for ccRCC, respectively, by the grid-search method [3]. The finalized hyperparameters after several trials are as follows: the DAE input layer loss rate and hidden layer loss rate are set to 0.1 and 0.3, and the $L_{1}$ and $L_{2}$ regularization are 0.005 and 0.1. The SAE activation and sparsity beta were set to 0.9 and 0.05, and the $L_{1}$ and $L_{2}$ regularizations were 0.003 and 0.1. The number of epochs is determined based on the reconstruction error for different numbers of epochs, which is finally determined to be 10. We selected 5 principal components for PCA and 3 independent components for ICA for each omic dataset in both simulation and real data analyses.

**2. Supplementary Note 2: Real datasets and data processing**

We focused on two independent cancer data: ccRCC and LGG. For ccRCC, we analyzed three types of omics data for each cancer: miRNA expression, mRNA expression, and promoter CpG methylation. Promoter CpGs were defined as methylation sites located exclusively within the promoter region, up to 2 kb upstream of the transcription start site [4], with those on sex chromosomes excluded. Three types of omics data were obtained, including miRNA expression data, mRNA expression data, and gene level methylation data. Features with a missing rate exceeding 30% in miRNA, mRNA, and methylation data were discarded, and the remaining missing values were imputed using the K-nearest neighbor (KNN) method [5]. To stabilize variance and reduce skewness, miRNA and mRNA expression data were subjected to a $\log_{2} \left( x+1 \right)$ transformation. For LGG patients, we included those with pathologic grades II and III and with available survival time (from initial diagnosis to death or the last follow-up) and survival status for further analysis. Three types of omics data were obtained, including miRNA expression data, mRNA expression data, and gene level methylation data, which were pre-normalized and quality-controlled by the data providers. Subsequently, we removed features with a coefficient of variation below the 50th percentile [6]. After preprocessing, we retained 388 miRNAs, 16,893 mRNAs, and 10,994 promoter CpG methylation features for 285 ccRCC patients, and 827 miRNAs, 19,416 mRNAs, and 14,470 methylated genes for 86 LGG patients. The baseline characteristics of ccRCC and LGG patients are presented in Tables S1.

**Table S1.** Baseline characteristics of ccRCC (left) and LGG (right) patients

| **Item** | **Classification** | ***n*(%)/mean ± SD** | |  | | **Item** | **Classification** | ***n*(%)/mean ± SD** |
| --- | --- | --- | --- | --- | --- | --- | --- | --- |
| Age, years |  | 59.98±10.58 |  | | | Age, years |  | 38.56±11.60 |
| Gender | Male | 185(64.91) |  | | | Gender | Male | 46(53.49) |
|  | Female | 100(35.09) |  | | |  | Female | 40(46.51) |
| Pathological stages | I | 138(48.42) |  | | | Tumor grade |  |  |
|  | II | 29(10.18) |  | | |  | II | 52(60.47) |
|  | III | 64(22.46) |  | | |  | III | 34(39.53) |
|  | Ⅳ | 54(18.95) |  | | |  |  |  |
| Survival outcome | Alive | 203(71.23) |  | | | Survival outcome | Alive | 44(51.16) |
|  | Dead | 82(28.77) |  | |  | | Dead | 42(48.84) |

Note: Categorical variables are presented as counts and percentages; while continuous variables are presented as mean±SD.

**3. Supplementary Note 3: Supplementary Table**

**Table S2. Clustering performance of CEDR and other methods assessed by ARI**

| **Method** | **SimData1 (Signal%=6%)** | | |  | **SimData2 (Signal%=8%)** | | |  | **SimData3 (Signal%=10%)** | | |
| --- | --- | --- | --- | --- | --- | --- | --- | --- | --- | --- | --- |
|  | **Low**  **noise** | **Moderate**  **noise** | **High**  **noise** |  | **Low**  **noise** | **Moderate**  **noise** | **High**  **noise** |  | **Low**  **noise** | **Moderate**  **noise** | **High**  **noise** |
| **CEDR** | 0.991  (0.035) | 0.931  (0.063) | 0.653  (0.106) |  | **0.999**  **(0.014)** | **0.975**  **(0.038)** | 0.807  (0.106) |  | **0.999**  **(0.016)** | **0.985**  **(0.046)** | 0.918  (0.068) |
| DAE-based | **0.987**  **(0.065)** | **0.936**  **(0.074)** | **0.673**  **(0.129)** |  | 0.972  (0.095) | 0.974  (0.066) | **0.840**  **(0.104)** |  | 0.972  (0.085) | 0.972  (0.089) | **0.923**  **(0.074)** |
| SAE-based | 0.990  (0.038) | 0.914  (0.08) | 0.603  (0.135) |  | 0.995  (0.043) | 0.972  (0.032) | 0.795  (0.126) |  | 0.992  (0.066) | 0.977  (0.065) | 0.909  (0.076) |
| PCA-based | 0.644  (0.175) | 0.455  (0.166) | 0.334  (0.040) |  | 0.769  (0.153) | 0.564  (0.181) | 0.381  (0.106) |  | 0.861  (0.139) | 0.650  (0.169) | 0.441  (0.159) |
| ICA-based | 0.335  (0.023) | 0.332  (0.022) | 0.320  (0.022) |  | 0.337  (0.024) | 0.334  (0.023) | 0.328  (0.023) |  | 0.336  (0.024) | 0.334  (0.023) | 0.330  (0.024) |
| COCA | 0.802  (0.184) | 0.671  (0.148) | 0.479  (0.156) |  | 0.805  (0.204) | 0.740  (0.181) | 0.594  (0.159) |  | 0.764  (0.215) | 0.761  (0.211) | 0.657  (0.149) |
| SNF | 0.525  (0.06) | 0.361  (0.048) | 0.263  (0.04) |  | 0.700  (0.082) | 0.453  (0.051) | 0.310  (0.043) |  | 0.853  (0.058) | 0.538  (0.06) | 0.353  (0.045) |
| CIMLR | 0.632  (0.066) | 0.429  (0.052) | 0.281  (0.045) |  | 0.796  (0.077) | 0.555  (0.061) | 0.351  (0.04) |  | 0.914  (0.045) | 0.646  (0.064) | 0.412  (0.051) |
| LSGMC | 0.577  (0.071) | 0.431  (0.039) | 0.324  (0.032) |  | 0.707  (0.095) | 0.495  (0.049) | 0.376  (0.036) |  | 0.805  (0.083) | 0.568  (0.075) | 0.418  (0.036) |
| MOSD | 0.457  (0.067) | 0.299  (0.056) | 0.166  (0.046) |  | 0.596  (0.061) | 0.383  (0.068) | 0.241  (0.048) |  | 0.699  (0.081) | 0.488  (0.062) | 0.285  (0.054) |
| PartIES | 0.886  (0.04) | 0.728  (0.051) | 0.429  (0.162) |  | 0.942  (0.032) | 0.815  (0.046) | 0.612  (0.086) |  | 0.971  (0.022) | 0.869  (0.043) | 0.694  (0.053) |

Note: The ARI values are presented as the mean (standard deviation) of 1,000 simulation runs. The best-performing result is highlighted in bold.

**Table S3. Clustering performance of CEDR and other methods assessed by AMI**

| **Method** | **SimData1 (Signal%=6%)** | | |  | **SimData2 (Signal%=8%)** | | |  | **SimData3 (Signal%=10%)** | | |
| --- | --- | --- | --- | --- | --- | --- | --- | --- | --- | --- | --- |
|  | **Low**  **noise** | **Moderate**  **noise** | **High**  **noise** |  | **Low**  **noise** | **Moderate**  **noise** | **High**  **noise** |  | **Low**  **noise** | **Moderate**  **noise** | **High**  **noise** |
| **CEDR** | 0.991  (0.02) | 0.933  (0.038) | **0.761**  **(0.072)** |  | **0.999**  **(0.009)** | **0.974**  **(0.025)** | 0.857  (0.052) |  | **0.999**  **(0.008)** | **0.986**  **(0.026)** | **0.924**  **(0.037)** |
| DAE-based | **0.987**  **(0.052)** | **0.935**  **(0.053)** | 0.734  (0.098) |  | 0.977  (0.074) | 0.972  (0.053) | **0.858**  **(0.068)** |  | 0.976  (0.064) | 0.974  (0.069) | 0.922  (0.053) |
| SAE-based | 0.988  (0.033) | 0.913  (0.059) | 0.684  (0.109) |  | 0.995  (0.035) | 0.967  (0.029) | 0.828  (0.085) |  | 0.993  (0.053) | 0.975  (0.054) | 0.907  (0.055) |
| PCA-based | 0.774  (0.121) | 0.632  (0.121) | 0.534  (0.04) |  | 0.854  (0.091) | 0.716  (0.13) | 0.573  (0.073) |  | 0.902  (0.079) | 0.777  (0.116) | 0.622  (0.114) |
| ICA-based | 0.46  (0.045) | 0.469  (0.048) | 0.466  (0.048) |  | 0.458  (0.045) | 0.464  (0.046) | 0.472  (0.046) |  | 0.454  (0.043) | 0.462  (0.047) | 0.472  (0.047) |
| COCA | 0.872  (0.125) | 0.781  (0.107) | 0.615  (0.119) |  | 0.867  (0.146) | 0.827  (0.126) | 0.716  (0.119) |  | 0.838  (0.157) | 0.838  (0.151) | 0.769  (0.111) |
| SNF | 0.573  (0.051) | 0.417  (0.044) | 0.311  (0.037) |  | 0.723  (0.062) | 0.506  (0.047) | 0.363  (0.041) |  | 0.849  (0.050) | 0.587  (0.051) | 0.407  (0.042) |
| CIMLR | 0.690  (0.052) | 0.494  (0.048) | 0.331  (0.046) |  | 0.823  (0.052) | 0.618  (0.054) | 0.412  (0.039) |  | 0.914  (0.036) | 0.708  (0.050) | 0.477  (0.046) |
| LSGMC | 0.666  (0.05) | 0.523  (0.045) | 0.379  (0.040) |  | 0.757  (0.062) | 0.593  (0.043) | 0.451  (0.044) |  | 0.824  (0.055) | 0.655  (0.052) | 0.506  (0.045) |
| MOSD | 0.532  (0.053) | 0.372  (0.044) | 0.198  (0.048) |  | 0.665  (0.051) | 0.461  (0.051) | 0.296  (0.044) |  | 0.758  (0.051) | 0.558  (0.054) | 0.357  (0.043) |
| PartIES | 0.895  (0.029) | 0.773  (0.034) | 0.493  (0.167) |  | 0.940  (0.026) | 0.840  (0.031) | 0.678  (0.083) |  | 0.967  (0.022) | 0.882  (0.029) | 0.748  (0.039) |

Note: The AMI values are presented as the mean (standard deviation) of 1,000 simulation runs. The best-performing result is highlighted in bold.

**Table S4. Clustering performance of CEDR and other methods assessed by ACC**

| **Method** | **SimData1 (Signal%=6%)** | | |  | **SimData2 (Signal%=8%)** | | |  | **SimData3 (Signal%=10%)** | | |
| --- | --- | --- | --- | --- | --- | --- | --- | --- | --- | --- | --- |
|  | **Low**  **noise** | **Moderate**  **noise** | **High**  **noise** |  | **Low**  **noise** | **Moderate**  **noise** | **High**  **noise** |  | **Low**  **noise** | **Moderate**  **noise** | **High**  **noise** |
| **CEDR** | **0.995**  **(0.029)** | 0.966  (0.052) | 0.742  (0.084) |  | **0.999**  **(0.012)** | **0.989**  **(0.03)** | 0.871  (0.103) |  | **0.999**  **(0.013)** | **0.991**  **(0.039)** | 0.958  (0.060) |
| DAE-based | 0.992  (0.051) | **0.971**  **(0.057)** | **0.776**  **(0.136)** |  | 0.983  (0.068) | 0.988  (0.05) | **0.914**  **(0.094)** |  | 0.984  (0.062) | 0.985  (0.069) | **0.965**  **(0.058)** |
| SAE-based | 0.995  (0.031) | 0.959  (0.062) | 0.712  (0.132) |  | 0.997  (0.038) | 0.989  (0.018) | 0.875  (0.115) |  | 0.993  (0.055) | 0.988  (0.051) | 0.957  (0.059) |
| PCA-based | 0.730  (0.13) | 0.604  (0.113) | 0.526  (0.040) |  | 0.824  (0.129) | 0.676  (0.127) | 0.560  (0.081) |  | 0.905  (0.123) | 0.735  (0.125) | 0.595  (0.109) |
| ICA-based | 0.551  (0.034) | 0.544  (0.036) | 0.531  (0.033) |  | 0.553  (0.037) | 0.548  (0.035) | 0.538  (0.036) |  | 0.553  (0.036) | 0.548  (0.035) | 0.539  (0.037) |
| COCA | 0.852  (0.138) | 0.756  (0.107) | 0.632  (0.114) |  | 0.860  (0.145) | 0.807  (0.133) | 0.707  (0.114) |  | 0.834  (0.149) | 0.827  (0.152) | 0.746  (0.107) |
| SNF | 0.729  (0.058) | 0.599  (0.047) | 0.527  (0.035) |  | 0.854  (0.061) | 0.672  (0.05) | 0.559  (0.040) |  | 0.939  (0.031) | 0.734  (0.058) | 0.594  (0.045) |
| CIMLR | 0.782  (0.067) | 0.644  (0.049) | 0.534  (0.038) |  | 0.899  (0.06) | 0.725  (0.059) | 0.584  (0.041) |  | 0.965  (0.021) | 0.790  (0.067) | 0.631  (0.047) |
| LSGMC | 0.745  (0.096) | 0.618  (0.057) | 0.527  (0.041) |  | 0.850  (0.091) | 0.672  (0.078) | 0.571  (0.046) |  | 0.911  (0.066) | 0.739  (0.099) | 0.607  (0.051) |
| MOSD | 0.655  (0.065) | 0.540  (0.059) | 0.458  (0.043) |  | 0.742  (0.076) | 0.604  (0.068) | 0.503  (0.048) |  | 0.817  (0.094) | 0.674  (0.061) | 0.529  (0.058) |
| PartIES | 0.953  (0.019) | 0.861  (0.051) | 0.638  (0.123) |  | 0.977  (0.013) | 0.916  (0.032) | 0.770  (0.076) |  | 0.989  (0.009) | 0.944  (0.023) | 0.835  (0.056) |

Note: The ACC values are presented as the mean (standard deviation) of 1,000 simulation runs. The best-performing result is highlighted in bold.

**Table S5.** Clinical and pathological characteristics of different subtypes in ccRCC

| **Characteristic** | **Cluster 1(n=115)** | **Cluster 2(n=63)** | **Cluster 3(n=70)** | **Outliers(n=37)** | **χ^2^/F** | ***P*** |
| --- | --- | --- | --- | --- | --- | --- |
| Age, years | 59.37±10.53 | 59.27±11.42 | 61.64±10.09 | 59.96±10.27 | 1.181 | 0.309 |
| Female, n (%) | 41(35.65) | 22(34.92) | 21(30.00) | 16(43.24) | 0.662 | 0.718 |
| Pathological stages |  |  |  |  | 23.232 | <0.001 |
| Grade I | 67(58.26) | 31(49.21) | 17(24.29) | 23(62.16) |  |  |
| Grade II | 10(8.70) | 6(9.52) | 6(8.57) | 7(18.91) |  |  |
| Grade Ⅲ | 23(20.00) | 13(20.63) | 25(35.71) | 3(8.11) |  |  |
| Grade Ⅳ | 15(13.04) | 13(20.63) | 22(31.43) | 4(10.81) |  |  |
| Death event, n (%) | 23(20.00) | 14(22.22) | 37(52.86) | 8(21.62) | 24.779 | <0.001 |

Note: The outlier group is shown for completeness but was not included in statistical comparisons. All statistical tests were performed among the three clusters (Cluster 1–3) only.

**Table S6.** Cox regression analysis of 248 ccRCC patients

| **Variables** | **Coefficient(SE)** | **Wald** | ***P*** | **HR (95% CI)** |
| --- | --- | --- | --- | --- |
| Subtypes |  |  |  |  |
| Cluster2 | 0.391(0.355) | 1.099 | 0.272 | 1.478(0.734-2.964) |
| Cluster3* | 0.908(0.284) | 3.204 | 0.001 | 2.480(1.423-4.323) |
| Age | 0.017(0.013) | 1.369 | 0.171 | 1.018(0.993-1.043) |
| Gender | -0.180(0.265) | -0.680 | 0.496 | 0.835(0.497-1.404) |
| Pathological stages |  |  |  |  |
| Grade II | 0.374(0.591) | 0.632 | 0.527 | 1.453(0.456-4.630) |
| Grade Ⅲ | 1.269(0.376) | 3.379 | 7.30E-04 | 3.558(1.704-7.430) |
| Grade Ⅳ | 2.013(0.355) | 5.664 | 1.48E-08 | 7.482(3.729-15.015) |

Note:*Shows statistically significant (*P* < 0.05). Cluster 1 was used as the reference group for subtype comparison, and Grade I was used as the reference for the comparison of differences between pathological stages.

**4. Supplementary Note 4: Characterization of excluded outliers in the ccRCC cohort**

To better characterize these excluded samples, we performed PCA visualization based on standardized mRNA expression profiles for illustration. The results show that the excluded samples do not form a compact or separable cluster but are instead sparsely distributed around the periphery of the main data cloud (see Figure S1). This pattern suggests that these samples represent heterogeneous aberrant deviations rather than a coherent biological subtype. Therefore, they were excluded from the primary clustering analysis to ensure the robustness of subtype identification.


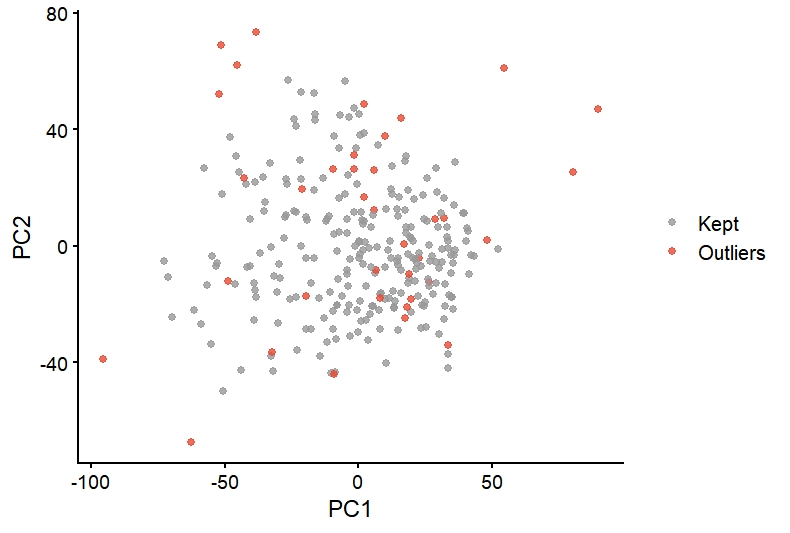


**Figure S1.** PCA of standardized mRNA expression profiles in the ccRCC cohort.

Alt text: A PCA plot of standardized mRNA expression profiles in the ccRCC cohort.

We have further characterized these outlier samples in terms of their clinical and survival features. As summarized in Table S5, the outliers are presented as an independent group alongside the three main subtypes. In addition, we incorporated them into the survival analysis as a fourth group (Figure S2). The survival curve of these outliers intersects with that of Cluster 2 and does not achieve the strong separation observed among the three main clusters. Therefore, excluding them from the primary clustering analysis is more biologically meaningful, as it allows for clearer prognostic stratification of the main subtypes.


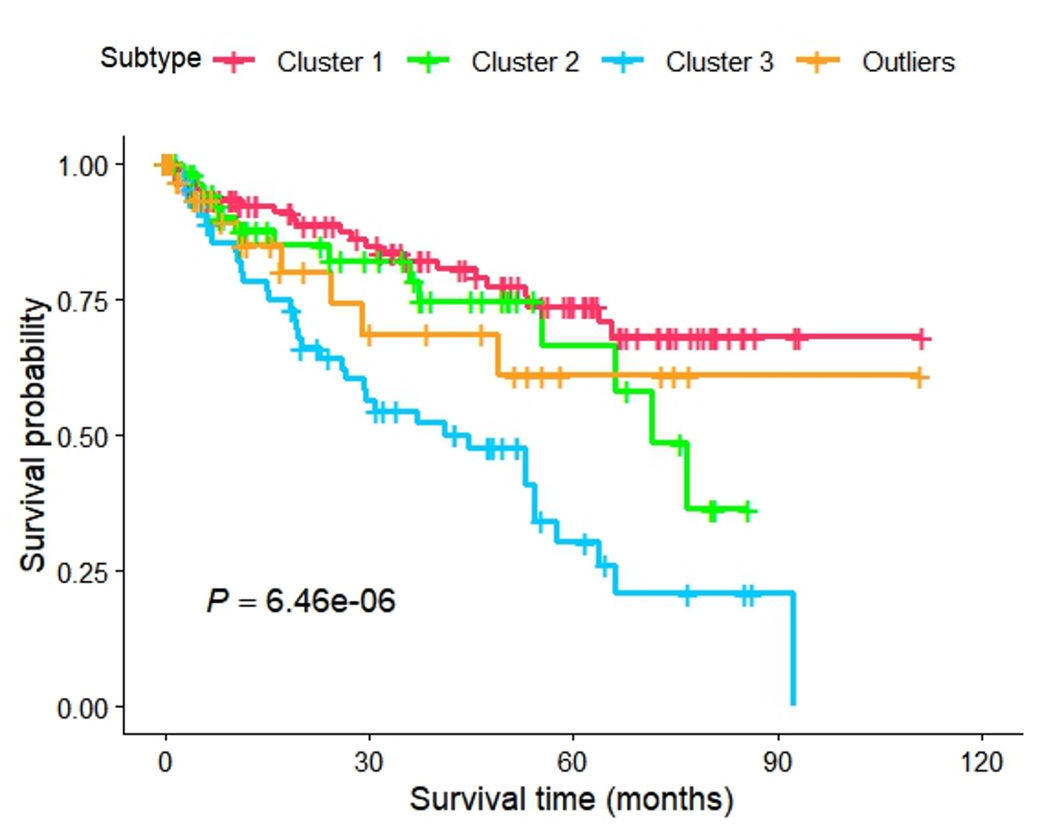


**Figure S2.** Kaplan-Meier survival curves of three molecular subtypes and outlier samples in ccRCC

Alt text: Kaplan-Meier survival curves showing three molecular subtypes and outlier samples in ccRCC.

**5. Supplementary Note 5: Real data analysis on LGG**

**5.1 Analysis of LGG subtypes identified by CEDR**

83 LGG patients were divided into two subtypes (53 patients in Cluster 1; 30 patients in Cluster 2) with significant differences in overall survival (see Figure S3A, $\chi^{2}=44.2$, log-rank *P*-value = 2.94 × 10⁻¹¹) using CEDR. 3 LGG patients were trimmed as outliers during OTRIMLE clustering to ensure the robustness of subtype identification. The relevant clinical data for each cluster were summarized in Table S7. In our subsequent analysis, we primarily focused on patients in Cluster 2, who exhibited a poor prognosis. Similarly, we fitted a multivariable Cox proportional hazards model to assess variations in prognosis among the subtypes. The results were summarized in Table S8, indicating that patients in Cluster 2 had a 7.724-fold higher risk of death compared to those in Cluster 1.

**Table S7.** Clinical and pathological characteristics of different subtypes in LGG

| **Characteristic** | **Cluster 1(n = 53)** | **Cluster 2 (n = 30)** | **χ^2^/t** | ***P*** |
| --- | --- | --- | --- | --- |
| Age, years | 36.92±9.71 | 42.03±13.88 | -1.784 | 0.081 |
| Female, n (%) | 25(47.17) | 14(46.67) | 0.002 | 0.965 |
| Tumor grade, n (%) |  |  | 66.97 | <0.001 |
| Grade II | 50(94.34) | 1(3.33) |  |  |
| Grade III | 3(0.57) | 29(96.67) |  |  |
| Death event, n (%) | 17(32.08) | 25(83.33) | 18.137 | <0.001 |

**Table S8.** Cox regression analysis of 83 LGG patients

| **Variables** | **Coefficient(SE)** | **Wald** | ***P*** | **HR (95% CI)** |
| --- | --- | --- | --- | --- |
| Subtypes |  |  |  |  |
| Cluster2* | 2.044(0.772) | 2.649 | 0.008 | 7.724(1.702-35.059) |
| Age | 0.013(0.013) | 1.016 | 0.310 | 1.013(0.988-1.039) |
| Gender | 0.078(0.318) | 0.245 | 0.806 | 1.081(0.580-2.017) |
| Tumor grade |  |  |  |  |
| Grade III | -0.233(0.775) | 0.301 | 0.764 | 0.792(0.173-3.619) |

Note: *Shows statistically significant (*P* < 0.05). Cluster 1 was used as the reference group for subtype comparison, and Grade II was used as the reference for the comparison of differences between tumor grades.

**5.2 Significant Feature Identification in LGG**

For LGG patients, differential expression analysis identified 4,402 DEmRNAs (1,948 downregulated and 2,454 upregulated), 24 DMGs (22 hypomethylated and 2 hypermethylated), and 56 DEmiRNAs (25 downregulated and 31 upregulated). Heatmaps illustrating DE across different omics data are presented in Figure S3B, with each row representing a distinct feature and each column corresponding to a patient. Red and green colors indicate relatively high and low expression levels, respectively, demonstrating heterogeneity between the two subtypes in LGG. A total of 694 overlapping genes were identified (Figure S3C).


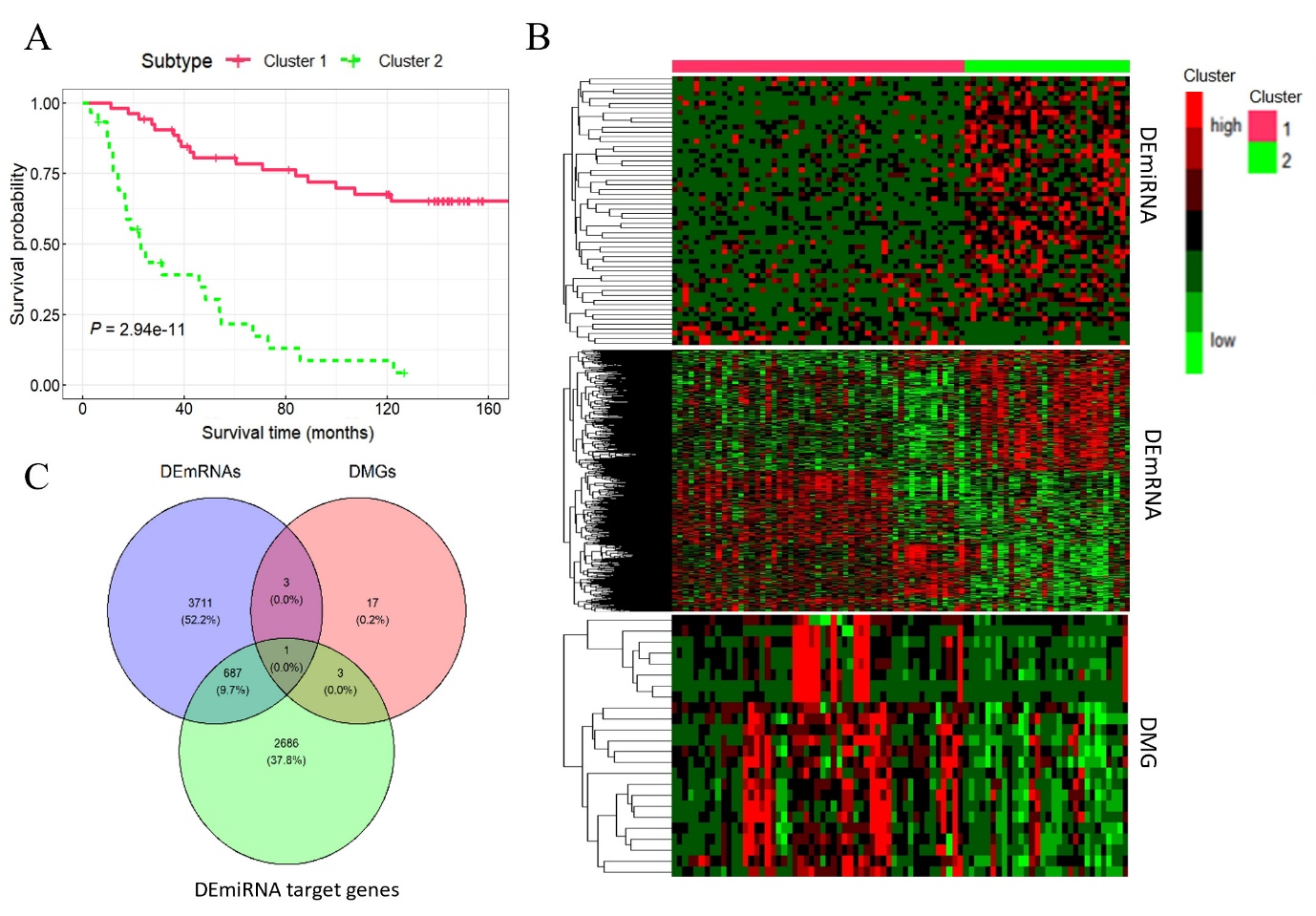


**Figure S3**. **Subtyping results of LGG.** (**A**)Kaplan-Meier curves of subtypes identified by CEDR for LGG. (**B**)The heatmaps of DEmRNAs, DEmiRNAs and DMGs between different clusters. (**C**)Venn diagram of differential genes in LGG patients.

Alt text: A multi-panel figure presenting LGG subtyping results, including survival curves, heatmaps of differential features, and a Venn diagram.

**5.3 GO and KEGG Pathway Enrichment Analysis in LGG**

For LGG, the top 10 enriched GO terms and KEGG pathways are presented in Figure S4. The GO terms were primarily enriched in the biological process categories of protein binding, cytosol, nucleus, cytoplasm, nucleoplasm, metal ion binding, plasma membrane, membrane, extracellular exosome, and positive regulation of transcription by RNA polymerase II. These results may reflect the presence of diverse glial cell types in the glioma microenvironment. In particular, protein binding has been implicated in glioma development; bioinformatics analyses have shown that PTB expression is significantly higher in glioma tissues than in normal tissues, and inhibition of protein-binding related genes may help delay glioma progression [7]. The KEGG pathways were enriched in metabolic pathways, pathways in cancer, axon guidance, human cytomegalovirus infection, proteoglycans in cancer, ErbB signaling pathway, regulation of actin cytoskeleton, Yersinia infection, hepatocellular carcinoma, and non-small cell lung cancer. Among these, the most significant pathways in LGG patients were metabolic pathways and axon guidance. Samad [8] mentioned that compared with normal cells, cancer cells adopt altered metabolic pathways, suggesting that dysregulation of energy metabolism may serve as a hallmark of cancer and provide a theoretical basis for clinical recognition.


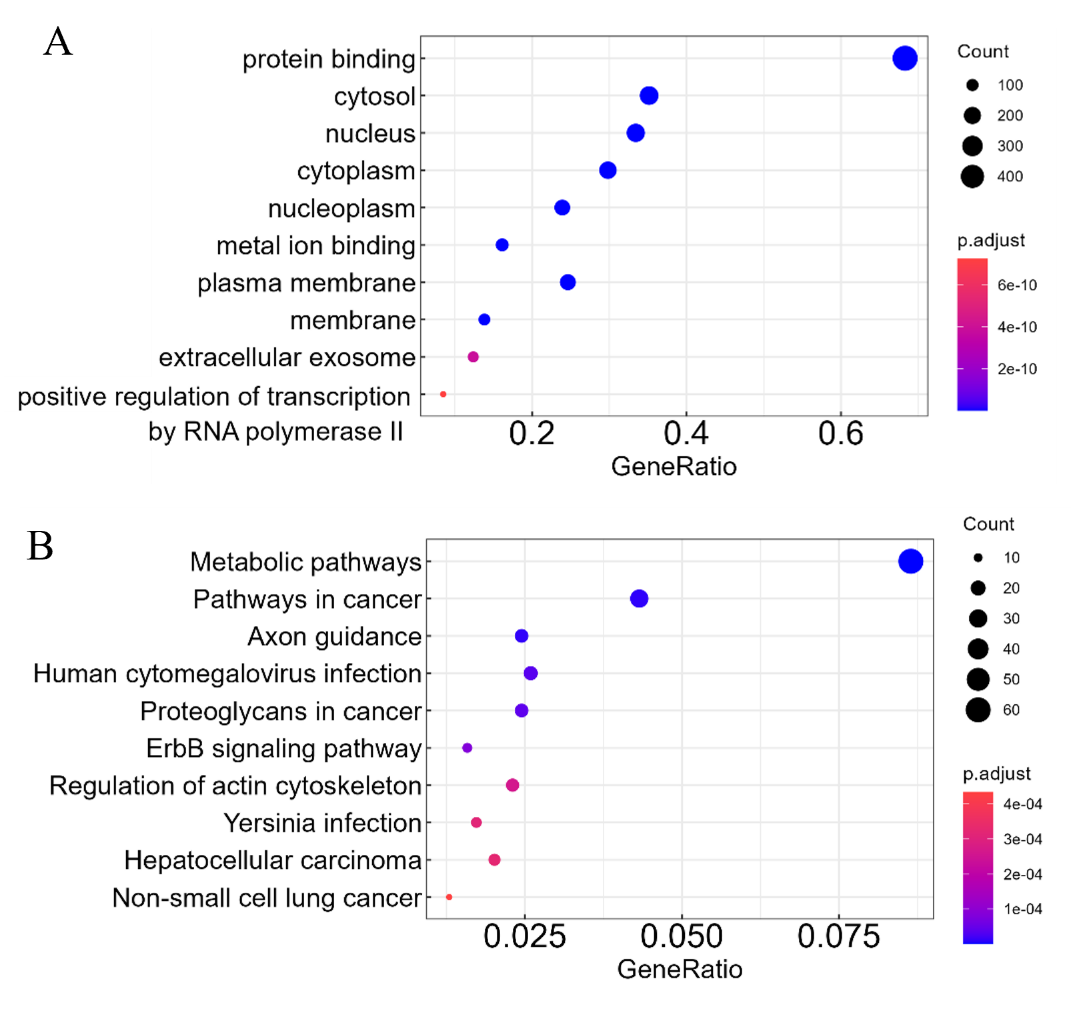


**Figure S4**. Enrichment Analysis of Overlapping Genes in LGG. (**A**) GO biological process enrichment and (**B**) KEGG pathway enrichment for 694 genes.

Alt text: Two enrichment plots showing Gene Ontology and KEGG pathway enrichment of overlapping genes in LGG.

**5.4 Immune cell** **infiltration analysis and pathway activity in LGG**

We also analyzed the pathway activity and immune infiltration of the LGG subtypes identified by CEDR. As shown in Figure S5A, we identified eight immune cell types exhibiting significant differences between the two subtypes: Common lymphoid progenitor, Mast cell, T cell CD4+ Th1, T cell regulatory, T cell CD8+ naive, Class-switched memory B cell, Macrophage, and Macrophage M1. Cluster 2 represents the subtype with the poorest prognosis in LGG, and its tumor microenvironment is characterized by high infiltration of Common lymphoid progenitor, T cell CD4+ Th1, T cell CD8+ naive, Macrophage, and Macrophage M1. In contrast, the abundance of Mast cell, T cell regulatory, and Class-switched memory B cell is relatively low. Studies have found that the level of mast cells increases in glioma samples, especially in high-grade gliomas. Mast cells can make glioma cells secrete macrophage attraction factors, leading to the increase of glioma-associated macrophages, and promote tumor proliferation, survival and migration. Therefore, mast cells are considered to be an important part of tumor microenvironment [9].

The pathway activity analysis of the two LGG subtypes identified three pathways with significant differences. As shown in Figure S5B, the Androgen pathway exhibited the highest activity in Cluster 1, while the EGFR and VEGF pathways were most active in Cluster 2. Androgen promotes glioma proliferation by binding to androgen receptors, triggering nuclear translocation and gene regulation [10] [11]. Targeting this pathway with AR inhibitors may delay tumor progression. EGFR activation induces CCL2 expression, enhancing glioma malignancy, drug resistance, and tumor-associated macrophage infiltration, highlighting its therapeutic potential [12]. Additionally, miR-376a directly inhibits SIRT1 expression in glioma cells, which in turn attenuates VEGF signaling, ultimately suppressing glioma cell proliferation [13].


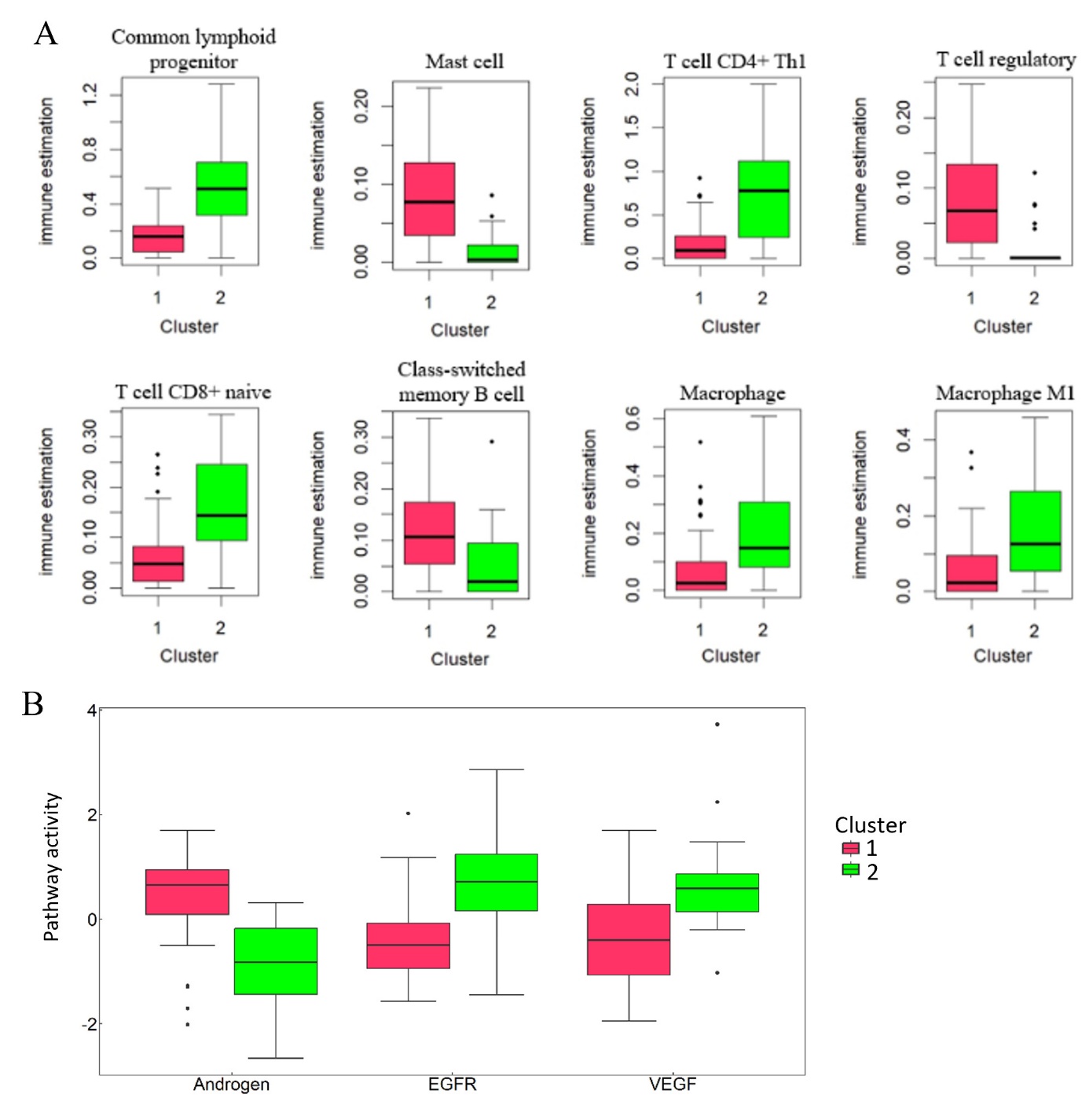


**Figure S5.** Differences in pathway activity and immune cell infiltration across clusters. **(A)** Abundance of Common lymphoid progenitor, Mast cell, T cell CD4^+^ Th1, T cell regulatory, T cell CD8^+^naive, Class-switched memory B cell, Macrophage, and Macrophage M1. **(B)** Pathway activity of Androgen, EGFR, and VEGF across two clusters in LGG.

Alt text: Multiple boxplots showing differences in pathway activity and immune cell infiltration across clusters in LGG.

**6. Supplementary Note 6: Real data analysis on LIHC**

We also applied CEDR to molecular subtyping of multi-omics data in Liver Hepatocellular Carcinoma (LIHC) from The Cancer Genome Atlas Program (TCGA). We considered three omics data types of each cancer, namely miRNA expression, mRNA expression, promoter CpG methylation from TCGA. We employed the same data preprocessing steps. The data used for analysis contained 7,995 mRNAs, 232 miRNAs, and 7,147 methylation features for the 287 LIHC patients from TCGA.

The results indicate that 262 LIHC patients were divided into two subtypes with significant different prognoses (log-rank P-value = 3.47 × 10⁻²). The corresponding survival curves are illustrated in Figure S6. Twenty-five LIHC patients were identified as outliers and excluded to enhance the robustness of subtype identification during OTRIMLE clustering. These results further demonstrate the robustness of CEDR in identifying clinically relevant molecular subtypes and support its applicability across diverse cancer.

**
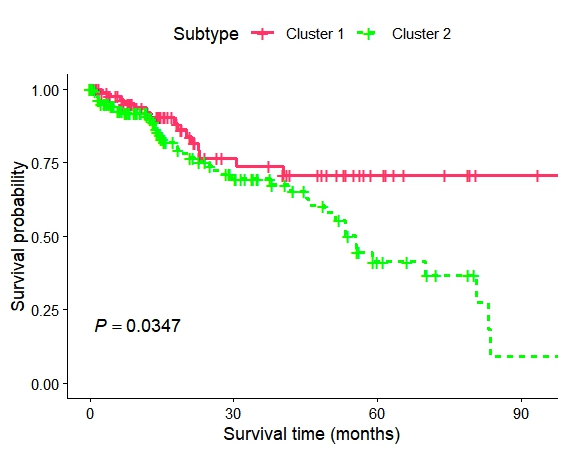
**

**Figure S6**. Subtyping results of 287 LIHC patients using CEDR

Alt text: Kaplan-Meier survival curves of LIHC patient subtypes using CEDR.

**7. Supplementary Note 7: Sensitivity analysis and Ablation Study**

**7.1 Sensitivity Analysis of DAE and SAE Hyperparameters**

The sensitivity analysis of the denoising autoencoder (DAE) was conducted under the SimData1 setting (Signal = 6%) with a high noise level ($\sigma^{2}$= 8). To avoid dominance by any single modality, subtype-specific signals were heterogeneously allocated across the mRNA, miRNA, and DNA methylation layers. The corruption rate of the DAE varied from 0.05 to 0.30, while the network architecture and the ensemble framework were kept fixed. For each corruption rate, the simulation experiment was repeated 1000 times to ensure the stability of the results. Across all tested corruption rates (see Figure S7), the resulting consensus clustering results showed only minor variation, indicating that the proposed framework is robust to moderate changes in denoising intensity. Based on this analysis, the corruption rate of the DAE was set to 0.1 in the main experiments.


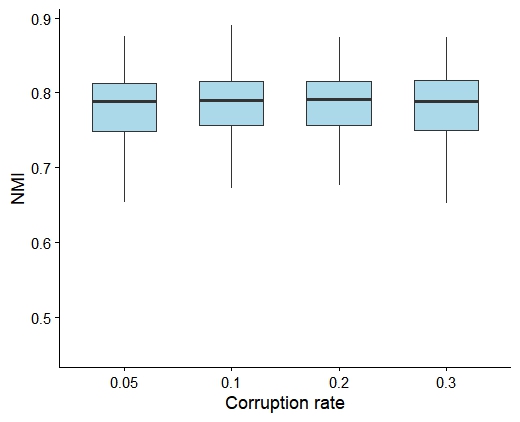


**Figure S7.** Sensitivity of clustering performance to corruption rate in the DAE

Alt text: Boxplots showing the sensitivity of clustering performance to corruption rate in the DAE.

To further evaluate the robustness of the SAE component, we conducted an extended sensitivity analysis on the sparsity hyperparameter (sparsity_beta), varying it from 0.01 to 0.20 while keeping the network architecture and other regularization parameters fixed. For each sparsity setting, the simulation experiment was repeated 1000 times. Across all tested sparsity levels, the consensus clustering results remained highly consistent across repeated simulations. Moreover, the corresponding NMI values exhibited only minor variation, indicating that variations in sparsity strength did not substantially affect the clustering structure (Figure S8). The sparsity parameter sparsity_beta was set to 0.05 in the main experiments.


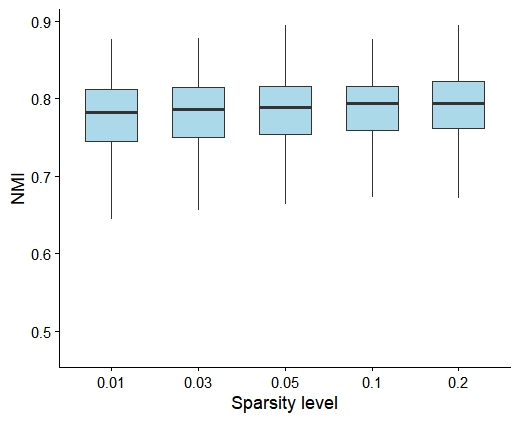


**Figure S8.** Sensitivity of clustering performance to Sparsity level in the SAE

Alt text: Boxplots showing the sensitivity of clustering performance to Sparsity level in the SAE.

**7.2 Ablation Study of Clustering Methods**

To assess the contribution of OTRIMLE to the robustness of our framework, we performed an ablation study based on our existing simulation setup. Specifically, we conducted 1000 independent runs under a scenario with noise ($\sigma^{2}$=4) and a signal proportion (Signal%=8%). After dimensionality reduction, we compared clustering results obtained using OTRIMLE, *k*-means, and spectral clustering. OTRIMLE consistently outperformed the other two methods and exhibited greater stability (see Figure S9). These results confirm that OTRIMLE is well-suited for robust clustering within our framework, as it simultaneously models principal components and potential outliers in DR-derived feature spaces, thereby better capturing the statistical properties of the reduced features.

**
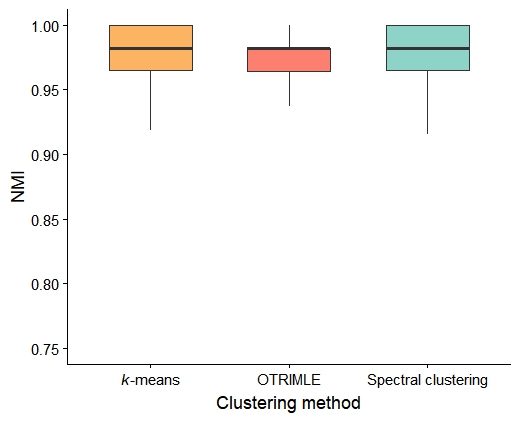
**

**Figure S9.** Performance Comparison of Clustering Methods

Alt text: Boxplots illustrating the comparison of clustering performance among k-means, OTRIMLE and spectral clustering.

**Reference**

1. Rifai S, Vincent P, Muller X et al. Contractive auto-encoders: Explicit invariance during feature extraction. In: Proceedings of the 28th international conference on international conference on machine learning. 2011, p. 833-840.
2. Karamizadeh S, Abdullah SM, Manaf AA. et al. An Overview of Principal Component Analysis, *J Signal Inf Process* 2013;04:173-175.
3. Kalian AD, Benfenati E, Osborne OJ. et al. Exploring Dimensionality Reduction Techniques for Deep Learning Driven QSAR Models of Mutagenicity. *Toxics* 2023;11:572.
4. Gusev A, Lee SH, Trynka G. et al. Partitioning Heritability of Regulatory and Cell-Type-Specific Variants across 11 Common Diseases. *Am J Hum Genet* 2014;95:535-552.
5. Troyanskaya O, Cantor M, Sherlock G. et al. Missing value estimation methods for DNA microarrays. *Bioinformatics* 2001;17:520-525.
6. Ren S, Fan A. K-means clustering algorithm based on coefficient of variation. In: 2011 4th International Congress on Image and Signal Processing. 2011, p. 2076-2079. IEEE.
7. Fontana L, Rovina D, Novielli C et al. Suggestive evidence on the involvement of polypyrimidine-tract binding protein in regulating alternative splicing of MAP/microtubule affinity-regulating kinase 4 in glioma. *Cancer Lett* 2015;359:87-96.
8. Samad A, Samant R, Venkateshwara Rao K et al. Oxaloacetate as a Holy Grail Adjunctive Treatment in Gliomas: A Revisit to Metabolic Pathway. *Cureus* 2023;15.
9. Chen R, Wu W, Liu T. et al. Large-scale bulk RNA-seq analysis defines immune evasion mechanism related to mast cell in gliomas. *Front Immunol* 2022;13:914001.
10. Daswani B, Khan Y. Insights into the role of estrogens and androgens in glial tumorigenesis. *J Carcinog* 2021;20:10.
11. Bao D, Cheng C, Lan X. et al. Regulation of p53wt glioma cell proliferation by androgen receptor-mediated inhibition of small VCP/p97-interacting protein expression. *Oncotarget* 2017;8:23142.
12. Zhou X, Liang T, Ge Y. et al. The crosstalk between the EGFR and IFN-γ pathways and synergistic roles in survival prediction and immune escape in gliomas. *Brain Sci* 2023;13:1349.
13. Deng Y-W, Shu Y-G, Sun S-L. miR-376a inhibits glioma proliferation and angiogenesis by regulating YAP1/VEGF signaling via targeting of SIRT1. *Transl Oncol* 2022;15:101270.

1. *Corresponding author: [caohy@sxmu.edu.cn](mailto:caohy@sxmu.edu.cn) (H. Cao), [cuiy@msu.edu](mailto:cuiy@msu.edu) (Y. Cui) [↑](#footnote-ref-1)
